# Design of PEGylated Three Ligands Silica Nanoparticles for Multi-Receptor Targeting

**DOI:** 10.3390/nano11010177

**Published:** 2021-01-12

**Authors:** Manon Maurel, Titouan Montheil, Julie Martin, Line Chaar, Veronica Guzman-Gonzalez, Morgane Couvet, Thibault Jacquet, Tao Jia, Beatrice Eymin, Karine Parra, Pascal Dumy, Jean Martinez, Florence Ruggiero, Elisabeth Vaganay, Ahmad Mehdi, Jean-Luc Coll, Gilles Subra

**Affiliations:** 1 Institute of Biomolecules Max Mousseron, Université de Montpellier, ENSCM, CNRS, 34095 Montpellier, France; manon.maurel@umontpellier.fr (M.M.); titouan.montheil@orange.fr (T.M.); julie.martin@umontpellier.fr (J.M.); karine.parra1@umontpellier.fr (K.P.); pascal.dumy@enscm.fr (P.D.); jean.martinez@umontpellier.fr (J.M.); 2 Institute for Advanced Biosciences, INSERM-UGA U1209, CNRS UMR 5309, 38700 La Tronche, France; line.chaar@univ-grenoble-alpes.fr (L.C.); veronica.guzman-gonzalez@univ-grenoble-alpes.fr (V.G.-G.); Morgane.couvet@inserm.fr (M.C.); thibault.jacquet2@gmail.com (T.J.); tao.jia86@gmail.com (T.J.); beatrice.eymin@univ-grenoble-alpes.fr (B.E.); 3 Institut de Génomique Fonctionnelle de Lyon, ENS de Lyon, UMR CNRS 5242, Université Lyon 1, 69364 Lyon CEDEX 07, France ; florence.ruggiero@ens-lyon.fr (F.R.); elisabeth.vaganay@ens-lyon.fr (E.V.); 4 ICGM, Université de Montpellier, ENSCM, CNRS, 34095 Montpellier, France

**Keywords:** silica nanoparticles, silylated peptides, sol–gel, fluorophore, surface functionalization, cancer targeting

## Abstract

The synthesis of silica nanoparticles (SiNPs) decorated on their surface with a range of various elements (e.g., ligands, drugs, fluorophores, vectors, etc.) in a controlled ratio remains a big challenge. We have previously developed an efficient strategy to obtain in one-step, well-defined multifunctional fluorescent SiNPs displaying fluorophores and two peptides ligands as targeting elements, allowing selective detection of cancer cells. In this paper, we demonstrate that additional level of controlled multifunctionality can be achieved, getting even closer to the original concept of “magic bullet”, using solely sol–gel chemistry to achieve conjugation of PEG chains for stealth, along with three different ligands. In addition, we have answered the recurrent question of the surface ungrafting by investigating the stability of different siloxane linkages with the ERETIC Method (Electronic Reference to Access In Vivo Concentrations) by ^19^F NMR quantification. We also compared the efficiency of the hybrid silylated fluorophore covalent linkage in the core of the SiNP to conventional methods. Finally, the tumor-cell-targeting efficiency of these multi-ligand NPs on human endothelial cells (HUVEC or HDMEC) and mixed spheroids of human melanoma cells and HUVEC displaying different types of receptors were evaluated in vitro.

## 1. Introduction

For the last decades, nanoparticles (NPs) have proven to be interesting tools for biotechnologies and biomedical applications, offering large possibilities in terms of diagnostics and drug delivery systems [1]. Silica NPs (SiNPs) are extensively used because of their simple synthesis by inorganic polymerization (sol–gel) process [2,3], their biocompatibility [4], tunable size, and capacity to encapsulate drugs or dyes [5]. Hopefully, at a low concentration of particles, silicates do no display toxicity [4,6]. However, the biocompatibility of SiNPs depends on their shape, charge, size, and bio-interface [7], and a perfect dovetailing of this parameter is still challenging. Thus, numerous strategies controlling the physicochemical properties of the nanoparticles were developed to modify their surface, thus improving their bioavailability, stability, and stealth [8,9]. Fluorescent NPs reach a major interest for in vivo bio-imaging [10], combining the potential of multifunctionalization and targeting with a fluorophore protecting effect [11,12,13]. Indeed, confining the dyes in a silica matrix enhances its chemical and physical stability, allowing for a longer conservation of the fluorescence in a biological media [14], thus protecting it from bleaching.

Herein, we present a one-pot synthesis of SiNPs-containing a dye, using the Stöber method, which yields a tailored and homogeneous size distribution around 100 nm [2]. For this study, Sulfo Cyanine 5.5 dye was chosen for its long emission wavelength in the near-infrared window to be used for cancer imaging [15]. To protect and enhance the stability of the dye, Cyanine was previously silylated to be covalently linked on the core of the silica matrix during the sol–gel process and not grafted to the surface after the SiNPs synthesis. This also allowed leaving more space on the surface for further grafting with one or more peptide ligands. For that purpose, we developed a versatile and efficient grafting strategy based on hybrid silylated peptides. First, peptides were selectively modified at one of their (the N- or C-terminal extremity) with a trialkoxysilane moiety. Hybrid peptides were then easily immobilized on the SiNPs surface by stable siloxane (Si-O-Si) bonds in one-step, under acidic conditions. In order to determine the grafting density and the absolute quantification of each ligand, ^19^F NMR analyses were performed. From these data, we could access to the putative conformation of the grafted biomolecules (i.e., mushroom and comb-like) on the SiNPs surface. ^19^F NMR allowed us to study the stability of the linkage confirming the stability of the siloxane grafting within the timeframes used for imaging and treatment. Finally, the impacts of ligand grafting, particle charge, and hydrodynamic radius were studied by Transmission Electron Microscopy (TEM), Dynamic Light Scattering (DLS), and zeta potential analysis.

Three different peptides were selected to prepare multifunctional antiangiogenic nanoparticles. The first peptide contains a cRGD motif recognizing integrin α_V_β_3_, a receptor overexpressed on the surface of activated endothelial cells. The second peptide, ATWLPPR, binds to Neuropilin 1, an important co-receptor of the Vascular Endothelial Growth Factor (VEGF). VEGF is one of the more potent angiogenic factor that stimulates the formation of neo-blood vessels. Both peptides were previously described [16,17]. Finally, the third chosen peptide, 23O (GTPGKOGPRGQRGPTGPRGERGP), contains the smallest fragment of HEPV, a polypeptide expected to block Fibroblast Growth Factor 2 (FGF2) proangiogenic activity [18]. However, in opposition with the two other peptides, 23O does not bind to a cell surface receptor but is expected to trap and block the soluble FGF2 growth factor, and thus to inhibit FGF2’s biological function. The combination of these three blocking peptides involved in distinct but redundant proangiogenic signaling pathways should ultimately provide a very powerful antiangiogenic nanoparticle.

A series of SiNPs grafted with different ratio of the three ligands were prepared and characterized. Their binding was measured on two different endothelial cell types which confirmed the efficiency of the ligand grafting strategy. At last, their variable reactivity and distribution in mixed tumor spheroids containing melanoma cancer cells and human endothelial cells were investigated, showing the influence of surface composition on their distribution.

## 2. Materials and Methods

Abbreviations: ACN, acetonitrile; Ac_2_O, acetic anhydride; APTES, 3-aminopropyltriethoxysilane; Boc, tert-butyloxycarbonyl; cRGD, cyclic Arg-Gly-Asp peptide derivative; DIEA, diisopropylethylamine; DMF, N,N′-dimethylformamide; DPBS, Dulbecco’s Phosphate-Buffered Saline; HATU,1-[Bis(dimethylamino)methylene]-1H-1,2,3-triazolo [4,5-b]pyridinium 3-oxid hexafluorophosphate; Fmoc, fluorenylmethoxycarbonyl; ICPTES, 3-isocyanatopropyltriethoxysilane; NRP, Neuropilin 1; NVOC, nitroveratyloxycarbonyl; O, Hydroxyproline; Pbf, 2,2,4,6,7-pentamethyIdlhydrobenzofuran-5-sulfonyl; PEG, poly(ethylene glycol); tBu, ter butyl; TEOS, tetraethylorthosilicate; TFA, trifluoroacetic acid; TFE, 2,2,2-trifluoroethanol; TIS, triisopropylsilane; TNBS, trinitrobenzenesulfonic acid; PS, polystyrene; rt, room temperature; SPPS, solid-phase peptide synthesis; Z, benzyloxycarbonyl, 23O, H-GTPGKOGPRGQRGPTGPRGERGP-NH_2_.

### 2.1. Synthesis of Hybrid Triethoxysilyl Sulfo Cyanine 5.5

A total of 10.1 milligrams (0.0091 mmol, 1 eq.) of Sulfo Cyanine 5.5 NHS ester was stirred in 300 µL of dimethyl sulfoxide containing 3.2 µL (0.014 mmol, 1.5 eq.) of 3-aminopropyltriethoxysilane (APTES), for 2 h, at room temperature, in the dark, to get hybrid Cyanine 5.5 called compound **1**. Hybrid fluorophore **1** was used as obtained in the next step (Supplementary Materials for hybrid triethoxysilyl sulfo Cyanine 5.5 for type **D**).

### 2.2. Synthesis of SiNPs (Example for Fluorescent SiNPs Type B)

Fluorescent silica nanoparticles were prepared by mixing 1.42 mL (36.46 mmol, 8.05 eq.) of a 28% ammonia solution with 25 mL of absolute EtOH. The DMSO solution containing hybrid fluorophore **1** was added (300 µL), followed by 1 mL of tetraethylorthosilicate (TEOS) (4.53 mmol). The reaction mixture was stirred at room temperature, for 24 h, in the dark. Fluorescent SiNPs were recovered by centrifugation, washed three times with EtOH and once with DPBS, and then conserved in DPBS, to prevent SiNPs aggregation. Then, 1 mL of the solution was washed with water twice and freeze-dried, to give a concentration of 10.8 mg/mL (Supplementary Materials for others SiNPs syntheses).

### 2.3. Cyanine Encapsulation Quantification (Example of Fluorescent SiNPs Type B)

Sulfo Cyanine 5.5 encapsulation was evaluated by UV absorbance quantification on a multi-label plate reader (Victor3 Multilabel Plate Reader, Perkin Elmer, Waltham, MA, USA). After dissolving blank SiNPs as control (1 mg) and fluorescent SiNPs (1.1 mg) in a 1.5 M NaOH solution (2.85 mL and 3.14 mL respectively), 200 μL of each solution was dropped on 6 wells of a 96-well plate reader, and measuring was performed. Absorbance was evaluated at 660 nm for the NaOH 1.5 M solution, blank, and fluorescent SiNPs.

### 2.4. Grafting of Hybrid Peptides on Fluorescent SiNPs (Example for **9**, **10** and **11** Hybrid PEG–Peptides, with a 1/1/1 Ratio)

A total of 1.852 mL of the fluorescent SiNPs type **B** solution was first centrifuged at 14.000 rpm, to remove DPBS excess. Then, 12.5 mg of hybrid PEG–peptide **9** (3.9 μmol), 14.4 mg of hybrid PEG–peptide **10** (4.1 μmol), and 12.2 mg of hybrid PEG–peptide **11** (2.4 μmol) were dissolved in 0.7 mL of an acidic aqueous solution (pH 2) (Supplementary Materials Figure S1–S3 for respectively PEG-peptide **9**, **10** and **11** syntheses). The PEG–peptides solution was then added to the SiNPs and stirred overnight, at 65 °C. Then, 10 mL of water was added, the solution was centrifuged at 7.800 rpm, and the filtrate was removed. This washing procedure was repeated twice with water (10 mL) and DPBS (10 mL). Then, SiNPs were suspended in DPBS (5 mL). Yield was estimated by ^19^F NMR, from an aliquot (1.5 mL) of the suspension that was centrifuged, washed twice with 1 mL of pure water, and then poured into 1 mL of pure water before being freeze-dried.

### 2.5. Grafting Quantification by ^19^F NMR (Example of **SiNP 7**)

Approximately 5 mg of **SiNP 7** grafted with 3 ligands was dissolved in 600 µL of 1.5 M NaOD/D_2_O under sonication. After hydrolysis of the tridimensional network of SiNPs, hybrid peptides and hybrid fluorophore were released, giving a characteristic yellow color of the cyanine dye in a basic media. The sample was then analyzed by ^19^F NMR (376 MHz, ^19^F). A standard solution of 0.05 mM TFA NaOD (1.5 M) in D_2_O was prepared as reference for the Electronic Reference to Access In Vivo Concentrations (ERETIC) method.

### 2.6. Anchoring Stability Study (Example for Type **B2**)

A total of 2.777 mL of the fluorescent SiNPs solution (10.38 mg/mL) was first centrifuged at 14.000 rpm, to remove DPBS excess. Then, 36 mg of hybrid PEG **2** (16 μmol) was dissolved in 0.7 mL of a 1%DMF/AcOH solution (*v*/*v*). The PEG solution was then added to SiNPs previously centrifuged, and the mixture was re-suspended before being stirred overnight at 65 °C. Then, 10 mL of DMF was added, and the solution was centrifuged at 7.800 rpm; then the filtrate was removed. This washing procedure was repeated twice, and then twice with EtOH (10 mL) and once with DPBS (10 mL). Then, the SiNPs were kept in suspension in DPBS (5 mL). A total of 800 µL of this suspension was collected every 24 h and centrifuged at 14.000 rpm. The supernatant was removed, and pure water was added to wash the SiNPs. This process was repeated twice. Supernatant was removed again, and 1 mL of pure water was finally added on SiNPs. After sonication and vortexing, the resulting SiNPs suspension was freeze-dried. The powder was weighted, dissolved in 600 µL of 1.5 M NaOD/D_2_O, and analyzed by ^19^F NMR. This procedure was repeated at day 1, 2, 3, 4, 6, and 14.

### 2.7. Cell Culture

Primary Human Umbilical Vein Endothelial Cells (HUVEC; Lonza, France, #2519A) and Human Dermal Microvascular Endothelial Cells (HDMEC; Lonza, France, #2516) were cultured in full EGM-2 or EGM-2 MV BulletKit media, respectively. Cells were cultured on plates coated with 1.33 mg/cm^2^ Collagen I, at 37 °C, in a 5% CO_2_ humidified atmosphere. Human melanoma M21 cells (kind gift of Dr. J. Gravier) were maintained in culture in a complete RPMI GlutaMAX^TM^ medium containing 10% (*v*/*v*) fetal bovine serum (previously inactivated for 30 min at 56 °C). The three cell lines expressed both αvβ3 integrin and Neuropilin 1 receptors.

### 2.8. Flow Cytometry (FACS)

The cells were harvested with 0.05% trypsin and then centrifuged for 5 min at 1200 rpm. After rinsing with PBS containing 1 mM of Ca^2+^ and Mg^2+^, 0.5 M cells per condition were incubated under agitation for 30 min in an NPs solution diluted in PBS, at the desired concentration (0.0025 mg/mL), at 37 °C, protected from light. Afterwards, cells were rinsed with PBS and centrifuged for 5 min at 1200 rpm. The cell pellet was resuspended in 500 μL of PBS, and the cells were analyzed by flow cytometry, using an Accuri c6 (BD biosciences).

### 2.9. Spheroids Binding Assay

Spheroids were generated by plating a mix of M21 (2000 cells/well) and HUVEC (2000 cells/well) into 96-well round-bottom ultra-low-attachment (ULA) spheroid microplates (Corning, Tewksbury, MA, USA), together with 0.01 mg/mL of nanoparticles in a culture medium made of 50% M21/50% HUVEC culture media, supplemented with penicillin (100 U/mL) and streptomycin (100 µg/mL), at 37 °C, in a humidified atmosphere with 5% CO_2_. Spheroids formation and growth were assessed by microscopic examination, using an inverted microscope.

### 2.10. Microscopy

Fluorescence microscopy was carried out by using a confocal microscope (LSM 710) with the Zen software (Carl Zeiss, Jena, Germany). Nanoparticles were observed by using a 10×/0.3 EC Plan-Neofluar objective lens after excitation at 633 nm with an emission filter from 640 to 740 nm. Except for the control, a z-stack was recorded for each spheroid (1 image each 8 µm of thickness), and images presented showed only one confocal plane located around the center of the spheroid. All images were acquired in the same experimental conditions. Because of the width of the different spheroids, 4 images were collected and assembled in the presented mosaic images, excepted in the presence of SiNP type **B** that produced compacted small spheroids.

## 3. Results

### 3.1. Synthesis and Characterization of Cyanine-Containing Fluorescent Nanoparticles

#### 3.1.1. What Is the Best NP Synthesis Procedure to Avoid Fluorophore Degradation? Stöber or Micro-Emulsion?

**Figure 1:**
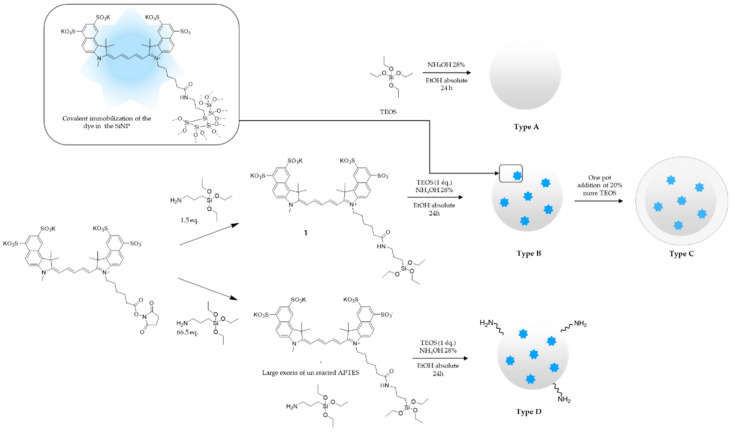
Preparation of SiNPs presenting different surface states. Type **A**, blank SiNPs; type **B**, fluorescent SiNPs obtained from hybrid cyanine **1**; type **C**, silica-coated type **B** SiNPs; type **D**, fluorescent SiNPs obtained with an APTES excess.

Our first attempts to prepare Fluorescent SiNPs were based on reverse water-in-oil microemulsion-mediated metallic alkoxide hydrolysis method [19,20], using cyclohexane, ammonia, hexane, and Triton X-100, with co-condensation between TEOS and our hybrid fluorophore **1** (0.2% molar). First, the triethoxysilylated fluorophore was prepared by reaction of commercially available Sulfo Cyanine 5.5 NHS ester with 3-aminopropyltriethoxysilane (APTES) to yield hybrid cyanine **1** in DMSO (Figure 1). It is noteworthy that we only used a 0.5 APTES excess, to limit the introduction of unreacted amino functions into the silica particle.

The surfactant was added to cyclohexane, under vigorous stirring. Then an ammonia solution (4.6 mmol/L) was added dropwise, followed by hexanal, to form the reverse micelles. Finally, the solution containing compound **1** and TEOS was added, to start the formation of fluorescent SiNPs. Unfortunately, and by contrast to what was observed with fluorescein containing SiNPs, a significant bleaching of the fluorophore occurred during preparation of the SiNPs, yielding greenish/brownish particles. We hypothesized that both concentrated ammonia and the use of surfactant facilitated the formation of free hydroxide ions, which attacked the π-conjugated system of cyanine, inducing degradation of the fluorophore. This side reaction was already reported by Lian et al. [13]. To overcome this problem, the surfactant-free Stöber method was essayed to synthesize the multi-ligand fluorescent SiNPs.

The solution containing hybrid compound **1** was poured into a mixture of concentrated ammonia (28%, 1.42 mL) and absolute ethanol (25 mL). Tetraethylorthosilicate (TEOS) was then added, to initiate the co-condensation with the hybrid fluorophore (0.2% molar), to give a theoretical number of 30,735 cyanine/SiNPs. By contrast to the reverse microemulsion process in which the size of the NPs was determined by the surfactant, the quantity of reagents and the reaction time were key factors for the Stöber synthesis [2]. We set up a 24 h reaction to successfully get ~100 nm diameter fluorescent SiNPs. Moreover, the absence of surfactant improved the stability of the fluorophore, as blue SiNPs were obtained. Summing up, we found the Stöber method superior to the microemulsion protocol to preserve Cyanine 5.5 fluorophore integrity.

#### 3.1.2. How Many Fluorophores Are Trapped in SiNPs?

To quantify the amount of Cyanine 5.5 encapsulated in the silica matrix, UV–vis titration was performed. First, a calibration curve was made by using NaOH 1.5 M solution, a blank SiNPs to mimic the presence of silica, and Cyanine 5.5 at different concentrations (Supplementary Materials Figure S4). The absorbance was plotted as a function of the Cyanine 5.5 concentration. This calibration curve was further used to calculate the quantity of encapsulated fluorophore. SiNPs were dissolved in a 1.5 M NaOH solution, to release the trapped fluorophore, and then the absorbance of the solutions was measured. After reporting absorbance values on the calibration curve, the number of mol of Cyanine 5.5/g of SiNPs (mol/g), and thus the number (N) of Cyanine 5.5 per SiNPs (N/NP), was evaluated (Table 1).

Whatever the synthesis protocol used to prepare fluorescent SiNPs (i.e., B, C, or D detailed in Section 3.2.1), the yield for encapsulation of the dye was around 20% (∼7 μmol/g) [21], around 7000 fluorophore/NP, which is enough to be detected in the biological assays. The different methods of fluorophore encapsulation seemed to have no significant influence on the encapsulation yield, even when a loss of around 1000 fluorophore/NP was observed between type **B** and type **C/D**. This could be explained by a thicker layer of silica in the case of type **C,** resulting in a longer release process of the fluorophore for quantification. For type **D** NPs, some degradation of the fluorophore during the silylation process was observed and might explain the difference of cyanine/NP number.

#### 3.1.3. How to Avoid Aggregation? Conservation of SiNPs

After centrifugation and washing, SiNPs were kept as a suspension in DPBS. This method of conservation was extremely important. Indeed, our first attempts to isolate NPs as a dry powder resulted in aggregated clusters (Supplementary Materials Figure S5). Consequently, to choose and adjust the desired concentration of SiNPs in assays, a small known volume was first collected, washed, and freeze-dried and weighted, allowing the determination of the original stock SiNPs solution in DPBS.

### 3.2. Influence of the Surface of the SiNPs for Grafting a Silylated PEG

#### 3.2.1. Synthesis and Characterization of SiNPs with Different Surfaces

The surface of the nanoparticles had to be decorated, and we proposed to first investigate the available surface on fluorescent NPs depending on the synthesis protocol. Four types of SiNPs were synthesized (Figure 1). Type **A** were blank SiNPs with no fluorophore; type **B** were fluorescent SiNPs prepared by following the two-step protocol described above (i.e., (i) preparation of the hybrid cyanine **1** with 1.5 eq. of APTES only and (ii) introduction of the hybrid fluorophore by the Stöber method); type **C** were type **B** NPs coated with an additional silica layer obtained by adding 20% more of TEOS for 6 h. At last, type **D** particles were similar to type **B** except that they were prepared according to a protocol, using a large APTES excess (APTES/TEOS theoretical ratio 13% molar) [22].

The sizes of all the four SiNPs were determined by TEM and DLS; zeta potential was measured at pH 7.6 (Table 1).

As expected, white and blue type **A** and **B** SiNPs were almost identical, with a diameter of about 100 nm determined by TEM and 130 and 145 nm by DLS, respectively. In the same way, their zeta potential was the same: −50 mV at pH 7.6. Thanks to the two-steps protocol we proposed, the introduction of a dye in the core of the particle did not affect the physicochemical properties of the NPs.

**Figure 2:**
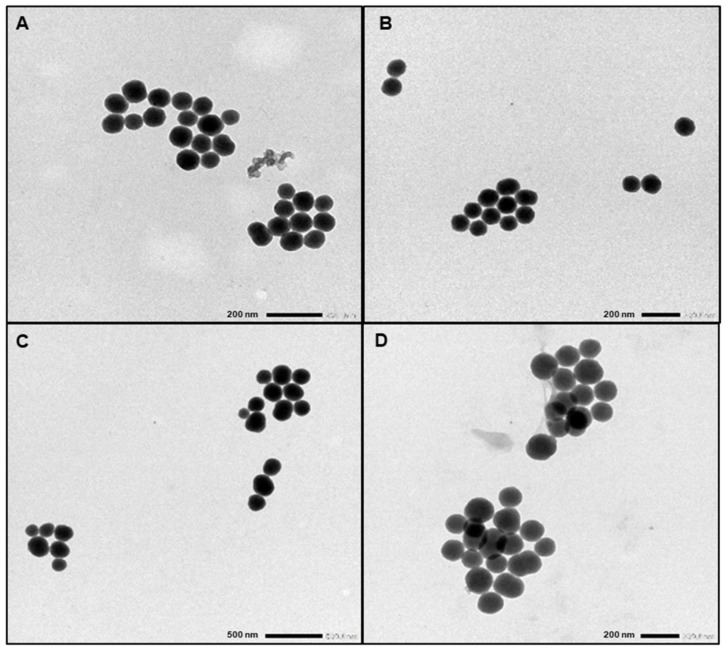
TEM analysis of SiNPs. Respectively, (**A**) type **A** SiNPs, (**B**) type **B** SiNPs, (**C**) type **C** SiNPs, and (**D**) type **D** SiNPs.

Type **C** particles were obtained from **B** particles by overlaid with an additional layer of silica. Consequently, and as expected, they were the biggest in the series, ranging from 160 to 210 nm diameter (Figure 2), as determined by TEM and DLS, respectively. Their zeta potential was −60 mV, in the same range than types **A** and **B**.

On the contrary, type **D** particles, obtained in the presence of a large APTES excess, differed greatly from their **A** and **B** counterparts. First of all, they aggregated, as witnessed by a higher value in DLS (515 nm) than the one obtained by TEM observation (135 nm) and a value of polydispersity index (PdI) of 0.5 (Table 1), showing a polydisperse population, as compared to the other types of SiNPs (PdI value range from 0.02 to 0.12) widely composed of monodisperse particles [23,24]. Aggregation was probably triggered by amino groups displayed at the surface, resulting from the large excess of APTES (i.e., 66.5 eq.) used during their preparation (theoretical ratio of 13% of amine compared to silicon atoms), which brought a low overall charge on the surface and induced a low electrostatic repulsion force [25]. Secondly, and besides aggregation, TEM examination clearly showed that type **D** NPs were still bigger (135 nm) than type **B** NPs (Figure 2). This could be explained by the APTES excess used during the synthesis, which led to a more basic medium accelerating the condensation process. This observation was supported by the green color of type **D** particles, as compared to the blue color of type **B** NPs, which could be related to a degradation of the dye, as observed with the microemulsion protocol (Supplementary Materials Figure S6) [13]. The UV/Visible spectra confirm a low degradation of the Cyanine 5.5 with a weak loss of absorption for type **D**, but the three fluorescent particles have still acceptable fluorescent intensity (Supplementary Materials Figure S7). Thirdly, and unsurprisingly, type **D** NPs presented a positive zeta potential value of +10 mV at pH 7.6.

In conclusion, these experiments show that the one-step protocol for type **B** SiNPs is the more relevant, with only poor excess of APTES and a good encapsulation yield, without affected the physicochemical characteristics of the surface.

#### 3.2.2. Is an Extra Shell of Silica Useful for Grafting? PEG Grafting on Type A, B, C and D SiNPs

**Figure 3:**
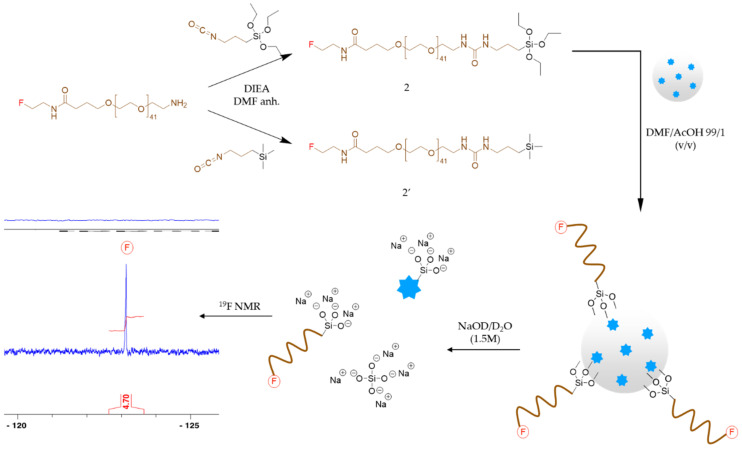
Up: Preparation of hybrid silylated and fluorinated PEG **2** and control molecule **2’**. Down: Preparation of PEGylated type **B** SiNPs from hybrid compound **2,** and the following quantification of fluorine by ^19^F NMR after dissolution of a NPs sample in 1.5 M NaOD solution (D_2_O).

Forming a siloxane bond is the most straightforward and easy way to graft organic compounds (e.g., peptides, dyes, drugs, and polymer chains) on a silica inorganic surface. By avoiding multistep conjugation reactions and being completely chemo selective towards other functional organic groups (e.g., alcohols, amines, carboxylic acids, guanidines, etc.), this approach is quicker to implement than using bioconjugations, which requires first modification of the silica surface with a suitable reactive group (e.g., azide, maleimide, thiol, alkyne, etc.). However, it requires the prior preparation of hybrid silylated precursors (e.g., hybrid peptides and hybrid PEG) usually obtained as alkoxysilane and chlorosilane derivatives. As a grafting model compound, we have prepared hybrid fluorinated, silylated PEG **2**, by first forming an amide bond between the carboxylic acid part of the commercially available Boc-NH-PEG_2000_-COOH and 2-fluoroethylamine. After removal of the Boc-protecting group in acidic conditions, the fluorinated PEG was silylated on his amine part, with an isocyanate derivative, leading to compound **2** (Figure 3). After hydrolysis, silanol condensation might occur in various experimental conditions, catalyzed either by pH or by nucleophiles such as fluoride ions [26]. To functionalize SiNPs, we used an optimized procedure, working at acidic pH. Indeed, in these conditions, hydrolysis of trialkoxysilyl precursors occurred quickly, while condensation in the solution was limited [27,28]. On the contrary, reaction in the presence of a high density of SiOH groups on the surface of the particle was favored over the in-solution homopolymerization of hybrid molecules.

We wanted to assess if the grafting could be hampered either by the presence of encapsulated fluorophore or even by the presence of amino groups resulting from an APTES excess. For that purpose, we reacted the four different surfaces of the four types of particles, with a PEG_2000_ silylated at one end, and bearing a fluorine atom at the other extremity (compound **2**, Figure 3). The grafted PEG was quantified by ^19^F NMR.

To achieve grafting, compound **2** (16 μmol) was solubilized in 700 μL of a solution of anhydrous DMF/AcOH 1% (*v*/*v*), then poured onto SiNPs previously centrifuged and free of supernatant. This mixture was re-suspended and stirred overnight, at 65 °C. Theoretically, this solution had a concentration of 42.8 mg/mL and 22.8 nM of compound **2**, for a theoretical result of 5.8 PEG/nm^2^. In such acidic conditions, hydrolysis of the triethoxysilane moieties occurred quickly, and unwanted condensation of silylated molecules in solution was prevented. On the contrary, reactions on the surface of the silica particles were favored, forming siloxane bond by nucleophilic attack of OH groups at the surface on the the R-Si(OH)_3_ in solution [29]. PEGylated particles were recovered by centrifugation, washed twice with 10 mL of DMF, twice with 10 mL of EtOH, once with 10 mL of DPBS, and then kept in 5 mL of DPBS. An aliquot (800 μL) of the PEGylated SiNPs suspension was taken, centrifuged, and washed twice with water, to eliminate DPBS salt residues. After washing, SiNPs were recovered in water, re-suspended, and then freeze-dried. The resulting SiNPs powder (4.4 mg) was dissolved in NaOD/D_2_O and analyzed by ^19^F NMR, to quantify free fluorinated PEG.

The sample concentration was determined by ERETIC method using standards containing a known concentration of TFA (0.1, 0.05, and 0.01 mM) in NaOD/D_2_O. The chemical shift expected for the fluorinated PEG (-NH_2_CH_2_CH_2_**F**) was at −123 ppm as a single peak. These data allowed us to calculate the number of PEG/nm^2^ for each type of particle, taking into account their surface depending on their diameter (Supplementary Materials for calculation). Results are gathered in Table 1.

We found that the efficiency of PEG grafting was almost the same for types **A** and **B** NPs regarding the number of PEG/nm^2^. About 3.5 PEG chain per nm^2^ were immobilized on the surface. However, type **C** SiNPs bearing an extra layer of silica enabled us to graft more than 6 PEG/nm^2^. This could be explained because the specific surface of re-coated SiNPs could be higher due to a more irregular morphology. However, one obvious explanation was that, for the same weight of silica, bigger particles displayed a lower surface than small ones. In other words, the theoretical accessible silica surface per gram of SiNPs, depends on the radius (Table 2). For example, for the same weight of SiNPs, the total accessible surface is 46% higher for 110 nm diameter particles than for the 160 nm diameter ones. In a reverse way, the same quantity of compound **2** (i.e., 0.5 µmol per mg of SiNPs) used for grafting represents a higher number of equivalents (about +46%) of reagent for type **C** SiNPs than for type **B** SiNPs. Taking into consideration the surface and normalizing the grafting on **A** NPs at 100%, **B** NPs reached 88%, **C** NPs 93%, and **D** NPs 67%. Summing up, adding an extra layer of silica allowed to get yields closed to that observed for non-fluorescent SiNPs, but this step was not necessary to obtain a good grafting yield. Conversely, we only obtained 67% of the grafting capacity for **D** SiNPs, indicating that amino groups hindered the surface.

At last, we also have performed a control experiment to clearly demonstrate the covalent nature of the bond between the hybrid silylated molecule and the SiNP surface. For that purpose, we prepared the fluorinated PEG molecule bearing a trimethylsilyl instead of a triethoxysilyl moiety (compound **2’** Figure **3**), which is theoretically unable to form any covalent bond with the silica. Using the same grafting procedure with compound **2’** on type **B** particles, we obtained no ^19^F signal after dissolving the particle, thus demonstrating that such a compound was not able to interact, even non-covalently, with the SiNP surface.

#### 3.2.3. What Is the Conformation of the PEG Chain on the Surface of the Particle?

**Figure 4:**
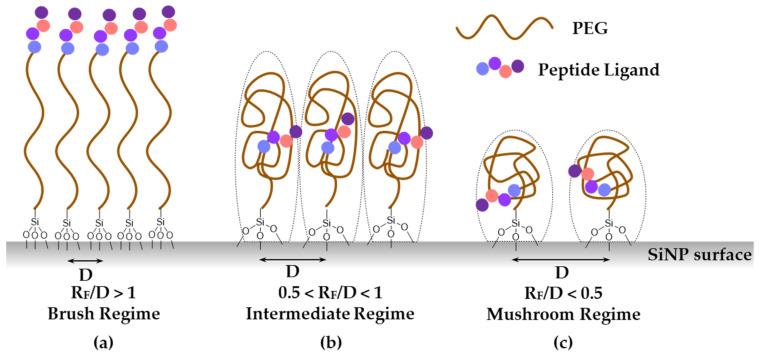
Schematic PEG–peptide behavior grafted on nanoparticle’s surface, depending on Flory’s radius R_F_ and distance between two anchoring points, D. (**a**), (**b**), (**c**) presentation for brush, intermediate and mushroom regime respectively.

PEGylation is a well-used strategy to stabilize biomolecules such as peptides and proteins in the bloodstream, thanks to its amphiphilic properties, which prevent enzyme-substrate hydrophobic–hydrophobic interactions [30] and thus decreases enzymatic degradation. Besides biomolecule conjugates, the addition of PEG chains on NPs is a common strategy to afford them stealth properties. Indeed, PEG-coated NPs also exhibit better bioavailability than non-coated ones [31], limiting opsonization, which triggers subsequent uptake by macrophages (i.e., capacity of antibodies to coat toxic antigens that can be recognized by phagocytic cells). The PEG corona modifies the hydrodynamic radius of SiNPs and leads to a zeta potential value closer to neutrality as the surface density increases and changes the accessibility to the anionic silica surface [32]. PEGylation also enhances potential cell–NP interactions when ligands are conjugated to the particle’s surface [33], because it allows for a good presentation of the ligands at the surface, particularly when the polymer chains are straight. Therefore, the conformation of the PEG chains at the surface of the particle is of outmost importance. Depending on the grafting density and their length, PEG chains may either “stand” close to each other in comb-like conformations or, on the contrary, crumble on the surface, forming mushroom-like structures inducing lower grafting densities. According to Adumeau et al. [34], PEG conformation on the surface of NPs (Figure 4) can be deduced from the PEG MW and grafting densities using the ratio R_F_/D. Where D is the average distance between two anchoring points and R_F_, the Flory’s radius that roughly corresponds to the space the PEG chain may occupy. Both can be calculated from Equations (1) and (2).

D = 2(πσ)−^1/2^ (1)

where σ is the value of surface grafting density (N/nm^2^), determined here by the ^19^F NMR analysis and the Flory’s radius R_F_:

R_F_ = αN^3/5^ (2)

where α is the effective monomer length = 0.358 nm for PEG, and N is the degree of polymerization, which is deduced from the PEG length [35].

If the ratio R_F_/D is greater than 1, the PEG will be in a brush conformation (a); with a value inferior to 0.5, the PEG will correspond to a mushroom conformation (c); and a value in between will correspond to an intermediate regime (b) (Figure 4).

To achieve a significant stealth behavior of SiNPs, the ratio R_F_/D must be noticeably superior to the mushroom–brush transition (R_F_/D ≧ 1), and so it exhibits a higher degree of PEG density, to avoid macrophage uptake [36]. The knowledge of the geometry of the PEG is of uttermost importance when targeting ligands have to be placed also on the surface. Indeed, placing peptide ligands at one of the extremity of the PEG chain does not guarantee their accessibility, unless the PEG is in a comb-like structure. Otherwise, the peptides might be hidden within PEG chains in the “mushroom” conditions, and might also fold back on the surface of the negatively charged silica surface.

Using our direct grafting method with silylated PEGs, we found that PEG corona was likely in a brush conformation, as we obtained a ratio R_F_/D greater than 1 for all the different SiNPs. For SiNP types **A**, **B**, and **D**, the ratio R_F_/D was approximatively 5.5, and for **C**, it was 7.2, indicating that they were in a brush regime (Table 1). In a brush regime, each PEG occupied less space on the surface of the SiNPs, and, consequently, the grafting density was higher than in a mushroom regime (Figure 4).

### 3.3. Is the Siloxane Bond between SiNP Surfaces and PEG Stable? Does the Nature of the Silane Moiety Impact on the Anchoring Stability?

**Figure 5:**
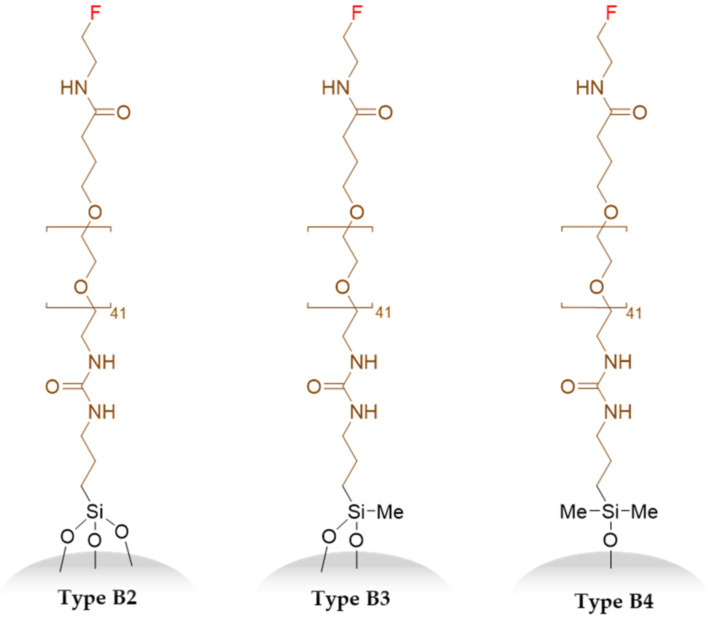
Fluorinated PEG particles (**B2**, **B3**, and **B4**) bearing three, two, and one anchoring points, respectively.

Silica-based materials are dynamic networks [37] which are degraded by hydrolysis and may rearrange through hydrolysis and condensation. It has been demonstrated that small particles with a higher surface area degrade faster [38]. Thus, it is legitimate to ask if the grafted elements on the SiNPs are not removed from the surface in physiological conditions at least during the timeframe they circulate in the blood stream for imaging or treatment purposes (two to three days). It is also of interest to know if the number of Si-O-Si bonds (i.e., the number of anchoring points) formed between a hybrid precursor and the silica (i.e., comparing R-SiMe(OH)_2_ with R-SiMe_2_(OH) and R-Si(OH)_3_) has an influence of the grafting stability (Figure 5).

To decipher these questions, three batches of type **B** SiNPs were functionalized with different silylated hybrid PEGs **2**, **3**, and **4** displaying, respectively, a triethoxysilane, a methyldiethoxysilane, and a dimethyl chlorosilane (Supplementary Materials). Grafting these precursors on the SiNP surfaces yielded, respectively, three, two, or a single Si-O-Si bonds between the PEG and the NP (Figure 5).

**Figure 6:**
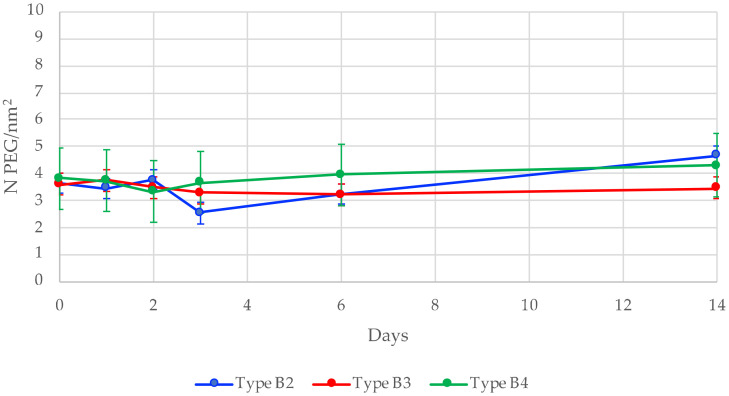
Grafting stability of **B2**, **B3**, and **B4** nanoparticles through time, with respectively three, two, and one anchoring points.

These particles (type **B2**, **B3**, and **B4)** were placed in DPBS (pH 7.6) for 14 days, to study the putative ungrafting of PEG chains by ^19^F NMR, using the ERETIC method (Figure 6).

At different time points at day 0, 1, 2, 3, 6, and 14, an aliquot of the suspension was taken. The SiNPs were centrifuged, the supernatant was removed, and the particles were washed twice with water and then freeze-dried before being dissolved in NaOD/D_2_O. ^19^F NMR quantification allowed us to determine the amount of PEG remaining on the surface of NPs, which was plotted as a function of time (Figure 6). The importance of centrifugation and washing steps has to be highlighted. They are necessary to avoid a potential re-grafting of silylated PEG molecules during the aliquot quantification that could have been released in DPBS during the experiment.

First, taking into account t = 0 measurements, we could claim that the type of silane chemistry did not have any influence on the grafting efficiency of the hybrid PEG. Indeed, the same grafting density was obtained for **B2**, **B3**, and **B4**, ranging from 3.6 to 4.6. Moreover, we observed that the grafting was stable even after two weeks. No significant decrease of grafted PEG at the surface was observed, confirming the stability of siloxanes (Si-O-Si) bonds linking the silica surface with the hybrid molecule. Last, the number of siloxane linkages had almost no influence on the stability of the grafted moieties. We could hypothesize that the numerous silanols present on the surface surrounding the grafted molecules could react with the hybrid moiety if it got hydrolyzed, thus reforming the SiOSi bond.

### 3.4. Design and Synthesis of Hybrid Ligands

Tumor cells overexpress different families of receptors at their surface, playing crucial roles in migration, differentiation, and survival of cells [39]. Surface functionalization of NPs with one or several ligands of those receptors can be viewed as a way to confer targeting properties to the nanovector, instead of relying only on the EPR effect. Grafting different ligands may also allow the targeting of heterogeneous tumors composed of cancer cells that do not always overexpress a given receptor. In addition, we have demonstrated that the multivalent presentation of one ligand by a single nanoparticle, as well as the simultaneous blockade of two different receptors at the surface of cancer cells by the presentation of two different ligands will induce the clustering of homo- and hetero-receptors responsible of the activation of complex signaling pathways [17].

In this study, we addressed the challenge to graft simultaneously three different hybrid ligands (compounds **9**, **10**, and **11**) on the surface of type **B** fluorescent SiNPs (Table 3 and Table 4).

Hybrid ligand **9** was based on the well-known integrin receptor antagonist cyclic peptide, i.e., c[RGDfK], that binds selectively with high affinity to α_v_β_3_ and α_v_β_5_ integrin receptors [17]. Hybrid ligand **10**, a silylated analogue of the antiangiogenic peptide H-ATWLPPR-OH, a neuropilin-1 receptor (NRP1) antagonist involved in cancer [40], was selected as the second ligand. The last ligand, **11**, came from a newly designed peptide named 23O, which was derived from HEPV and binds to the fibroblast growth factor-2 (FGF2) [18]. Ligands **9** and **10** were thus expected to bind cell surface receptors, while ligand **11** should capture the intracellular FGF2 growth factor. Combining the three activities should provide a powerful antiangiogenic activity in a large panel of solid tumors.

**Figure 7:**
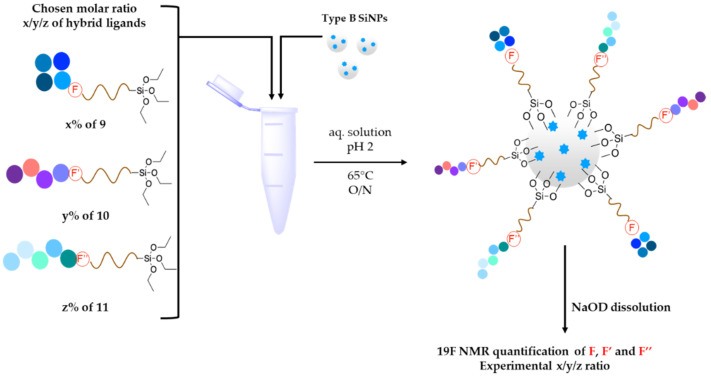
Preparation of multi-ligand fluorescent SiNPs with a simultaneous grafting of x/y/z ratio of hybrid ligands **9**, **10**, and **11**.

These molecules were designed on the same geometry: the peptide ligand that will be anchored on the SiNP surface was separated from the triethoxysilyl moiety by a PEG_2000_ spacer and elongated by a short fluorinated spacer consisting in three to five non-natural amino acids (i.e., β-Alanine and fluorinated amino acids, in red in Table 3). As already discussed, the PEG spacer allowed for a good presentation of the ligand at the NP’s surface when the polymer chains were in a brush regime. The fluorinated spacers were composed of β-Ala and three different fluorinated amino acids: 2-amino-4,4,4-trifluoro-butyric acid (AlaCF_3_) for compound **9**; 4-fluorophenylalanine (PhF) for compound **10**, and 4-(trifluoromethyl)phenylalanine (PhCF_3)_ for compound **11**. The purpose of these fluorinated residues was to provide distinctive ^19^F NMR signals, allowing their simultaneous quantification when they were present on the same SiNP. Indeed, each ligand displayed a different chemical shift: −64 ppm for **9**, − 117 ppm for **10**, and −62 ppm for **11** (noted F, F’, and F’’ in Figure 7, respectively). At last, a negative control of the c[RGDfK] cyclic peptide was also synthetized c[REGfK], to lead to compound **8** and its hybrid form **12**.

Syntheses of these hybrid multifunctional ligands shared common steps. Peptide ligands bearing the fluorinated spacer were prepared (peptides **5**, **6**, **7**, and **8**, Table 3) and purified before being coupled to the Boc-NH-PEG_2000_-COOH spacer, and finally silylated to yield hybrid compounds **9**, **10**, **11**, and **12**.

However, dedicated strategies were followed for the preparation of the ligand parts (Supplementary Materials for detailed procedures). Briefly, cyclic peptide **5** was synthesized by fragment coupling. First, H-Asp(OtBu)-(*D*)Phe-Lys(Z)-Arg(Pbf)-Gly-OH was synthesized on solid support and then cyclized in solution to yield c[ArgGlyAsp(D)PheLys] after deprotection. The fluorinated spacer Fmoc-βAla-Ala(CF_3_)-[βAla]_2_-OH was synthesized on solid support. The two fragments were coupled in solution using the ε-NH_2_ of Lys of the cyclic peptide and the C terminus carboxylic acid of the spacer to obtain peptide **5**. Its negative control compound **8**, followed the same synthetic pathway than the linear sequence H-(*D*)Phe-Lys(Z)-Arg(Pbf)-Glu(OtBu)-Gly-OH.

Linear peptides **6** and **7** and the N-terminus spacer sequence were synthesized stepwise on solid support. All side chain protecting groups were TFA-labile and removed before the silylation step with the notable exception of the Lysine residue within the sequence of peptide **7** that had to remain protected during the reaction with ICPTES. For that purpose, we used the Nvoc (*o*-nitroveratryloxycarbonyl) photolabile protecting group, which could be cleaved in mild conditions by irradiation after silylation, without affecting the triethoxysilane moiety, thus avoiding unwanted premature hydrolysis and condensation.

After purification of peptides **5–8**, Boc-PEG_2000_-COOH was coupled by forming a peptide bond between the carboxylic acid of the PEG and the N terminus of the fluorinated spacer linked to the peptide ligand. At last, the Boc-protecting group was removed, using TFA/DCM (50/50 *v/v*), and the free primary amine was then reacted by using ICPTES to form a urea bond. The resulting hybrid ligands were used for grafting, without further purification (Supplementary Materials).

### 3.5. Hybrid Ligand Grafting

We planned to prepare nine batches of different particles (Table 4) displaying various combinations of the three ligands. SiNPs **1**, **2**, and **3** displayed a single type of ligand at their surface (ligands **9**, **10**, and **11,** respectively); SiNPs **4**, **5,** and **6** were particles with two ligands coated with an approximatively 50/50 ratio of each hybrid ligand: **9** + **10** for compound **4**, **10** + **11** for compound **5**, and **9** + **11** for compound **6**. SiNP **7** was a mix of the three ligands. Finally, SiNPs **8** and **9** were negative controls bearing, respectively, the ligand **2** for compound **8** and the non-binding analogue of the RGD sequence, i.e., the hybrid ligand **12** for compound **9**.

As explained, our method allowed for the simultaneous grafting of the hybrid ligands on type **B** SiNPs. The amount of each of the three hybrid ligands was adjusted (Supplementary Materials) to get a 15 μM total concentration, which corresponded to a large excess of ligands relatively to the accessible silica surface. Indeed, 20 mg of particles was introduced, corresponding to a total surface of 5.10^17^ nm^2^. Assuming there was one peptide, each square nanometer indicated that about four equivalents were used, which corresponded to a high grafting density. Noteworthy, our preliminary attempts using a DMF/AcOH 1% (*v*/*v*) solution for grafting hybrid peptides **9**, **10**, and **11** proved to be ineffective, with only 0.7% of the hybrid peptides present in solution being actually grafted. We then modified our procedure by using aqueous 0.01 M of HCl (pH 2). At this pH, the hydrolysis rate was high, while condensation was minimal. These experimental conditions avoided intermolecular condensation and promoted heterogeneous condensation, reaching about 6% grafting yield.

Grafting proceeded as follows. First, seven batches of 1.852 mL of SiNP type **B** (10.8 mg/mL) in DPBS were centrifuged, to remove the supernatant. In parallel, aqueous solutions at pH 2 (1.633 mL) of each hybrid PEG–peptide (around 15 nM) were prepared. Centrifuged SiNPs (20 mg) were poured in 700 µL of one solution for a single ligand grafting, or poured in a mixture of 350 µL of two solutions for a dual ligand grafting, or poured in a mixture of 233 µL of three solutions for the triple-ligand grafting (Figure 7). SiNPs were re-suspended and stirred overnight, at 65 °C. The grafted SiNPs were then washed twice with water and once with DPBS, and then kept in DPBS solution (5 mL). An aliquot of this solution was washed twice with water and then freeze-dried, dissolved in NaOD/D_2_O, and used for ^19^F NMR quantification.

Quantification results are gathered in Table 4. We first noticed that total peptide grafting densities were in the same range, varying from 0.11 to 0.98 hybrid peptide per NPs square nanometer. Interestingly, this value was lower than the grafting density of 3.6 obtained with hybrid PEG **2** only (SiNP **8**). This could indicate that the peptide sequence placed at the top of PEG_2000_ exerted a significant influence on the grafting probably by steric but also by a charge–repulsion effect. Indeed, at pH 2, all the peptides of hybrid compounds **9–12** were positively charged. Accordingly, PEG–peptides were likely in a brush regime with results of R_F_/D above 1, and therefore they were well presented at the NPs’ surface for targeting purpose.

The grafting with a single ligand gave about 0.5 PEG–peptide/nm^2^. A lower grafting density (0.2 PEG–peptide/nm^2^) was obtained for ligand **11**. This could be explained by a longer peptide chain (twice than the others) and a higher positive charge at pH 2 (four protonation sites in the 23O peptide vs. only one for peptides **5** and **6**). When mixing two hybrid ligands together in the grafting solution, it is known that the grafted ratio roughly corresponds to the concentration ratio in the solution [16]. As expected, the experimental ratio of **9**/**10** grafting in SiNP **4** corresponded to theory. This was not the case for hybrid ligand **11**. However, the **9**/**11** and the **10**/**11** ratio measured for both SiNP **5** and SiNP **6** matched with the theory. Surprisingly, the PEG–peptide **11** was overrepresented compared to **9** and **10**. Ligand **9** was grafted with a lower efficiency, compared to the two others. The length and/or the charge of the hybrid compound in the solution could have an important impact on the yield of the grafting reaction.

Furthermore, the grafting seems to have no strong impact on the fluorescence intensity, and grafted SiNPs are still sufficiently fluorescent (Supplementary Materials Figure S8).

**Figure 8:**
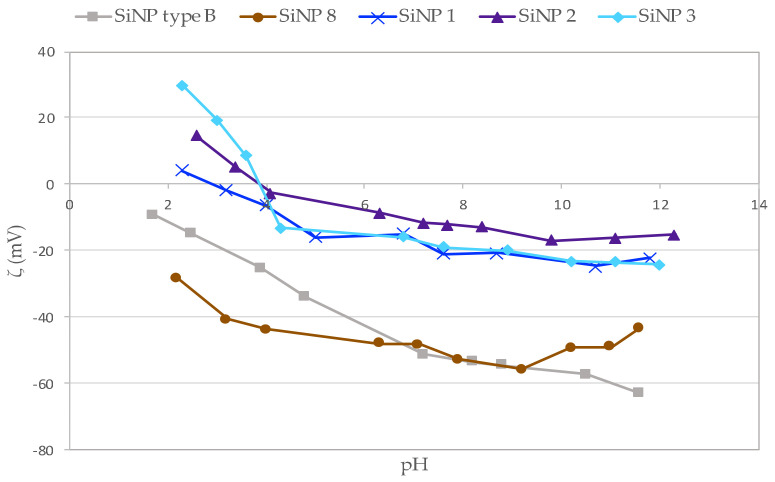
Zeta potential as a function of pH of SiNPs type **B** (in gray), SiNPs with PEG **2** (in red), SiNPs with PEG–peptide **9** (in blue), compound **10** (in purple), and compound **11** (in dark blue).

Zeta potential studies were performed on type **B** SiNPs, and on mono ligand nanoparticles SiNPs **1–2-3** (Table 4). Each batch of SiNPs conserved in DPBS solution was diluted in Milli-Q water to obtain a final concentration of 0.1 mg/mL. This dilution step (nearly 100 times for SiNP type **B** and 30 times for SiNPs with ligands) was crucial to decrease the phosphate concentration, which could lead to unreliable zeta measurement. Thereby, the water medium in excess had a low ionic strength (conductivity measured varying from 0.25 to 0.6 mS/cm for samples prepared in pure water) [34]. Then, the pH of each batch was adjusted (HCl or NaOH) at different concentrations (0.01M, 0.1M or 1 M), and measured (Figure 8). As expected for all particles, the higher the pH, the more negatively charged the surface of SiNPs. This was due to the formation of silanolate (Si-O^-^) ions at high pH. On the contrary, at low pH we expected SiNPs with less charges [41]. Results are reported in Figure 8.

As expected, non-functionalized type **B** SiNPs reached the lowest negative values. Indeed, their surface was composed of only SiOH moieties that were able to form a high number of negatively charged silanolates SiO^-^ at basic pH. PEGylated SiNP **8** shared roughly the same behavior, with less negative values and some SiOH moieties being engaged covalently to form SiOSi bonds with hybrid PEG chains. Whatever the conformation PEG chains, they might act as a screen for the charges at the SiNP’s surface [34]. Logically, when grafted with hybrid peptide–PEGs **9**, **10**, and **11** (blue curves), the resulting SiNPs behaved differently from type **B**, being mostly positively charged below pH 4 and displaying a zeta potential no lower than -22 mV, even in highly basic conditions (pH 12). Indeed, and as already discussed, peptides grafted at the end of the PEG chain presented protonation sites due to lysine and, more significantly, arginine side chains. Indeed, the guanidine side chain of arginine (pKa 12.5) was positively charged in a wide range of pH.

### 3.6. Binding Efficiency

**Figure 9:**
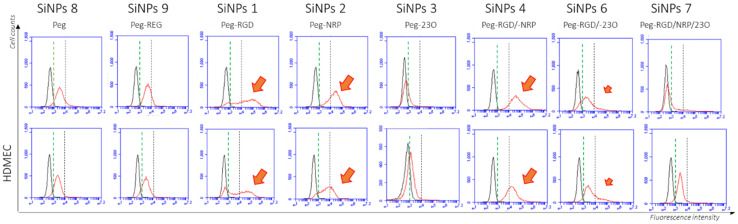
Binding efficiency on endothelial cells. HUVEC and HDMEC endothelial cells are expressing the α_v_ß_3_ and NRP1 receptors. The binding efficiency of each type of SiNP was evaluated by flow cytometry after 30 min incubation in suspension with the cells. *X*-axis: Log. Fluorescence intensities. *Y*-axis: cell counts. Vertical dotted lines: max level of autofluorescence of the cells (green); max level of non-specific labeling with the SiNPs (black). Orange arrows indicate the presence of positively labeled cells and their size indicates the population of positively stained cells.

The different types of nanoparticles **B 1** to **9**, were tested by FACS (flow cytometry) for their binding ability on two different endothelial human cell types expressing integrin α_v_ß_3_ and Neuropilin 1 receptors. As it can be seen in Figure 9, a very weak positive signal was obtained by using negative control nanoparticles SiNP **8** (PEG) and SiNP **9** (Peg–REG) resulting from non-specific interactions with the cells (fluorescence intensities ranging between 10^3^ and 10^4^). This signal was more important using SiNP **1** (Peg–RGD) and SiNP **2** (Peg–NRP), and negative with SiNP **3** presenting the 23O peptide. This pattern was expected, since both cell lines expressed the two cell surface receptors, while the 23O ligand was not designed to bind a membrane receptor. The 1/1 mixed SiNPs **4** and **6** (Peg–RGD/Peg–NRP and Peg–RGD/23O) presented decreased binding efficiency, as compared to SiNP **1** in which only the cRGD motif was present. Finally, combining the three peptides together resulted in no binding, suggesting that the number of RGD motifs, in particular, was too low on these NPs.

### 3.7. Cell Labeling and Interaction with Melanoma/Endothelial Mixed Spheroids

**Figure 10:**
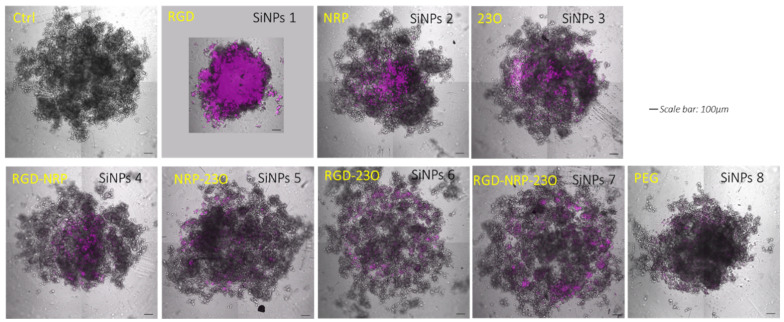
Binding efficiency on spheroids containing M21 human melanoma cells plus endothelial cells. Both M21 melanoma and HUVEC endothelial cells express the a_v_ß_3_ and NRP1 receptors. The binding efficiency of each type of SiNPs on the mixed spheroids was evaluated by fluorescence microscopy after excitation at 633 nm and with an emission filter from 640 to 740 nm, using a 10x objective.

We then investigated the binding and impact of the presence of these nanoparticles on mixed melanoma/HUVEC spheroids. In these experiments, human M21 melanoma and HUVEC cells were mixed and grown in suspension, in the presence of 0.01 mg/mL of the different nanoparticles. One day later, the spheroids were forming aggregates of cells, covering an average diameter of 1 to 1.5 mm (Figure 10). Using a confocal fluorescent microscope, we could detect the presence of the different nanoparticles. SiNPs **1** (RGD–nanoparticles) strongly labeled the totality of the spheroids, but, more importantly, their presence led to a two-fold compaction of the cell aggregates. This suggested that the multivalent presentation of the RGD motif stimulated and tightened the cell–cell contacts. By contrast, SiNPs **2** (NRP–nanoparticles) were only located in the center of the spheroids, and less numerous than SiNPs **1**. However, their fluorescence was significantly higher when compared to both the control and SiNPs **8** (PEG–nanoparticles). SiNPs **3** (23O–nanoparticles) accumulated less efficiently in the spheroids and were homogenously dispersed. The different combinations of two or three peptides neither increased the intensity of labeling, nor did it induce the specific compaction observed with RGD-only nanoparticles. As expected, these results demonstrated the significant binding of all types of nanoparticles, except the PEG ones, to M21/HUVEC mixed spheroids. Importantly, their contact with the cells in 3D induced different phenotypes on the spheroids that will have to be further investigated regarding the presented ligands, as well as their possible antiangiogenic activities in vivo in tumor-bearing animals.

## 4. Conclusions

In this paper, we have described a generic method to design well-controlled multi-ligand fluorescent SiNPs, using the sol–gel process. The key feature of this synthesis was the use of hybrid PEG–peptides bearing one trialkoxysilane moiety, which enabled their grafting on the silica surface through Si-O-Si linkage, in acidic conditions. This methodology allowed the controlled simultaneous grafting of three peptides. To establish the reliability of this type of particle, we have optimized the grafting procedures and studied the dye incorporation, the grafting efficiency, the PEG–peptide conformation, and the anchoring stability.

We have shown the efficiency of these nanomaterials in preliminary in vitro tests, in particular on cancer spheroids, highlighting clearly the influence of the ligands anchored to the surface of the particles. The potential interest of the combination of the three different peptides should be further studied in vivo in mice bearing solid tumors. In particular, it will be interesting to use these tools to investigate their antiangiogenic activity as a function of the type and ratio of each peptide, as well as the capacity of these multifunctional particles to be active in heterogeneous tumors that might not always present the three proangiogenic targets.

Besides this approach, the generality of our process (sol–gel synthesis and siloxane bond functionalization) opens the gate to a wide range of multifunctional nanomaterials for diagnostic, as well as theragnostic applications, with porous SiNPs.

## Figures and Tables

**Table 1:** Size (TEM and DLS), zeta potential (**ζ**), quantification of hybrid PEG **2** grafting, and fluorophore encapsulation of the different SiNP types.

Type of SiNPs	Size (TEM) (nm)	Size (DLS) (nm)	PdI	Zeta Potential (ζ) at pH 7.6 (mV)	Hybrid PEG Loading (μmol/g)	N PEG/nm^2^	R_F_/D	Conformational Regime	Cyanine 5.5 Incorporation (yield%)
**A**	90	130	0.12	−47	170	3.37	5.4	Brush	n.a.
**B**	110	145	0.09	−50	149	3.62	5.6	Brush	23
**C**	160	210	0.02	−60	170	6.03	7.2	Brush	17
**D**	135	510 ^a^	0.50	+10	110	3.28	5.3	Brush	19

PdI = polydispersity index; n.a. = not applicable. ^a^ Aggregation was observed by TEM.

**Table 2:** Accessible silica surface and theoretically vs. experimentally number of mol of PEG/nm^2^.

Type of SiNPs	Total Area/g (nm^2^/g)	Max Theoretical μmol of PEG/nm^2^	Experimental μmol of PEG/nm^2^	Yield of Grafting ^a^ (%)
**A**	3 × 10^19^	1.8 × 10^−17^	5.5 × 10^−18^	31.8
**B**	2.5 × 10^19^	2.2 × 10^−17^	6 × 10^−18^	28
**C**	1.7 × 10^19^	3.4 × 10^−17^	1 × 10^−17^	29.5
**D**	2 × 10^19^	2.5 × 10^−17^	5.5 × 10^−18^	21.5

^a^ Yield calculated by comparing the quantity of compound **2** in solution and the resulting quantity grafted.

**Table 3:** Peptides **5–8** and corresponding hybrid ligands **9–12.**

Compound # Ligand/Hybrid Ligand	Ligand MW	Ligand m/z Found	Ligands R = H Hybrid Ligands R = 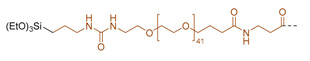	^19^ F δ (ppm)	Peptide Isoelectric Point (IP)
**5/9**	955.4	956.9 [M+H]^+^	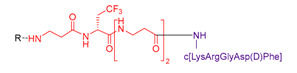	−64	7.2
**6/10**	1228.7	645.35 [M+2H]^2+^	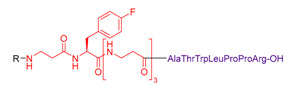	−117	11.1
**7/11**	2879.4	961.6 [M+3H]^3+^	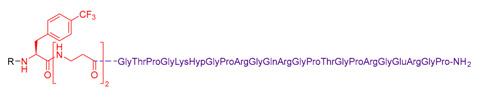	−62	12.5
**8/12**	969.5	970.8 [M+H]^+^	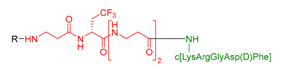	−64	7.4

**Table 4:** Relative and quantitative integration of ^19^F NMR signals of NPs grafted with PEG–peptides **9**, **10**, and **11**, and negative control (PEG and PEG–peptide **12**).

SiNP	Theo. Ratio 9/10/11	Exp. Ratio ^a^ 9/10/11	Overall Theoretical Peptide Loading (µmol/g)	Overall NMR Peptide Loading (µmol/g)	N PEG–Peptide/nm^2^	Zeta Potential (ζ) at pH 7.6 (mV)	R_F_/D ^b^
SiNP _1_	100/0/0	100/0/0	207	26	0.6/0/0	−20	2.3
SiNP _2_	0/100/0	0/100/0	196	20.2	0/0.5/0	−12	2.0
SiNP _3_	0/0/100	0/0/100	128	8.2	0/0/0.2	−19	1.3
SiNP _4_	49/51/0	55/45/0	198	7.4	0.9/0.08/0	/	0.9/0.8/0
SiNP _5_	0/63/37	0/8/92	161	7.8	0/0.01/0.1	/	0/0.3/1.2
SiNP _6_	62/0/38	37/0/63	165	13.2	0.1/0/0.2	−2	1.0/0/1.3
SiNP _7_	38/39/23	7/70/23	178	11.2	0.01/0.2/0.06	/	0.4/1.3/0.7
SiNP _8_	/	/	242	149	3.62	−48	5.6
SiNP _9_	/	/	219	18	0.4	/	1.9

^a^ R_F_/D ratio was calculated by using the number of PEG–peptide/nm^2^ obtained by ^19^F NMR and the value given for R_F_ of PEG_2000_ only, assuming that peptides did not significantly affect the Flory’s radius. ^b^ Determined by Electronic Reference to Access In Vivo Concentrations (ERETIC) ^19^F NMR.

## Data Availability

Not applicable.

## Supplementary Materials

The following are available online at https://www.mdpi.com/2079-4991/11/1/177/s1. Figure S1: Synthesis of hybrid PEG–peptide 9: c[Asp-DPhe-Lys[(EtO)_3_Si(CH_2_)_3_NHCO-NH-PEG_2000_-βAla- Ala(CF_3_)-βAla_2_)]-Arg-Gly]. Figure S2: Synthesis of hybrid PEG–peptide 10: (EtO)_3_Si(CH_2_)_3_NHCO-NH-PEG_2000_-βAla-Phe(4-F)-βAla_3_-Ala-Thr-Trp-Leu-Pro-Pro-Arg-OH. Figure S3: Synthesis of hybrid peptide 11: (EtO)_3_Si(CH_2_)_3_NHCOPhe(4-CF_3_)-βAla_2_-Gly-Thr-Pro-Gly-Lys- Hyp-Gly-Pro-Arg-Gly-Gln-Arg-Gly-Pro-Thr-Gly-Pro-Arg-Gly-Glu-Arg-Gly-Pro-NH_2_. Figure S4: Calibration curve for Cyanine 5.5 encapsulation. Figure S5: Aggregated dried SiNPs, Figure S6. type **B** vs. type **D** SiNPs. Figure S7: UV/Visible spectra of SiNPs types **B**, **C**, and **D**. Figure S8: UV/Visible spectra of SiNPs type **B**, SiNPs **1**, SiNPs **2**, and SiNPs **3**.
