# Supplementary material for: Design of PEGylated Three Ligands Silica Nanoparticles for Multi-Receptor Targeting
Source: Nanomaterials (Basel). 2021 Jan 12;11(1):177. doi: 10.3390/nano11010177 (PMC7828255; doi:10.3390/nano11010177)
Supplement: Supplementary file 1 [file nanomaterials-11-00177-s001.pdf]

nanomaterials

Supplementary materials

Design of PEGylated Three Ligands Silica Nanoparticles for Multi-Receptor Targeting

Manon Maurel [1], Titouan Montheil [1], Julie Martin [1], Line Chaar [2], Veronica Guzman-Gonzalez [2], Morgane Couvet [2], Thibault Jacquet [2], Tao Jia [2], Beatrice Eymin [2], Karine Parra [1], Pascal Dumy [1], Jean Martinez [1], Florence Ruggiero [3], Elisabeth Vaganay [3], Ahmad Mehdi [4,*], Jean-Luc Coll [2,*] and Gilles Subra [1,*]

[1] Institute of Biomolecules Max Mousseron, Université de Montpellier, ENSCM, CNRS, 34095, Montpellier, France; manon.maurel@umontpellier.fr (M.M.); titouan.montheil@orange.fr (T.M.); julie.martin@umontpellier.fr (J.M.); karine.parra1@umontpellier.fr (K.P.); pascal.dumy@enscm.fr (P.D.); jean.martinez@umontpellier.fr (J.M.)

[2] Institute for Advanced Biosciences, INSERM-UGA U1209, CNRS UMR 5309, La Tronche, France; line.chaar@univ-grenoble-alpes.fr (L.C.); veronica.guzman-gonzalez@univ-grenoble-alpes.fr (V.G.-G.); Morgane.couvet@inserm.fr (M.C.) ; thibault.jacquet2@gmail.com (T.J.); tao.jia86@gmail.com (T.J.); beatrice.eymin@univ-grenoble-alpes.fr (B.E.)

[3] Institut de Génomique Fonctionnelle de Lyon, ENS de Lyon, UMR CNRS 5242, Université Lyon 1, Lyon, France; florence.ruggiero@ens-lyon.fr (F.R.); elisabeth.vaganay@ens-lyon.fr (E.V.)

[4] ICGM, Université de Montpellier, ENSCM, CNRS, 34095 Montpellier, France

* Correspondence: ahmad.mehdi@umontpellier.fr (A.M.); jean-luc.coll@univ-grenoble-alpes.fr (J.-L.C); gilles.subra@umontpellier.fr (G.S.)

Citation: Maurel, M.; Montheil, T.; Martin, J.; Chaar, L.; Guzmán-Gonzalez, V.; Couvet, M.; Jacquet, T.; Jia, T.; Eymin, B.; Parra, K.; et al. Design of PEGylated Three Ligands Silica Nanoparticles for Multi-Receptor Targeting. *Nanomaterials* **2021**, *11*, 177. https://doi.org/10.3390/nano11010177

Received: 19 December 2020
Accepted: 9 January 2021
Published: 12 January 2021

Publisher's Note: MDPI stays neutral with regard to jurisdictional claims in published maps and institutional affiliations.

Copyright: © 2021 by the authors. Licensee MDPI, Basel, Switzerland. This article is an open access article distributed under the terms and conditions of the Creative Commons Attribution (CC BY) license (http://creativecommons.org/licenses/by/4.0/).

Abbreviations

ESI-MS: electrospray ionization mass spectrometry; HPLC: High Performance Liquid Chromatography; LC-MS: Tandem Liquid Chromatography/Mass Spectroscopy. Other abbreviations used were those recommended by the IUPAC-IUB Commission (Eur. J. Biochem. 1984, 138, 9-37).

General Experimental and Synthetic Procedures

The 2-chorotrityl chloride resin (100-200 Mesh, 1.60 mmol/g Cl/g resin) was purchased from Iris Biotech and stored at 4°C. Protected amino acids and HATU were purchased from Iris Biotech, Senn Chemicals and Bachem. (S)-Fmoc-2-amino-4,4,4-trifluorobutyric acid, Fmoc-4-trifluoro (L) methylphenylalanine and Fmoc-4 fluoro (L) phenylalanine were purchased from Iris Biotech, Sigma Aldrich and Interchim, respectively. Sulfo-Cyanine5.5 NHS ester was purchased from Lumiprobe and Boc-NH-PEG_{2000}-COOH from Iris Biotech (Marktredwitz, Germany).

All reagents and solvents were from AlfaAesar, Acros, Sigma-Aldrich or Merck (Kandel, Germany) and were used without further purification.

LC/MS Analyses

Samples for LC/MS analyses were prepared in an acetonitrile/water (50:50, v/v) mixture, containing 0.1% TFA. The LC/MS system consisted of a Waters Alliance 2695 HPLC, coupled to a Water Micromass ZQ spectrometer (electrospray ionization mode). All analyses were carried out using a Phenomenex Onyx, 25 mm × 4.6 mm reversed-phase column. A flow rate of 3 mL/min and a gradient of (0–100)% B over 2.5 min were used. Eluent A: water/0.1% HCO_2H; eluent B: acetonitrile/0.1% HCO_2H. Electrospray mass spectra were acquired at a solvent flow rate of 200 μL/min. Nitrogen was used as both the nebulizing and drying gas. The data were obtained in a scan mode ranging from 100 to 1000 m/z or 200 to 1600 m/z in 0.7 sec intervals; six scans were summed to obtain the final spectrum.

RP-HPLC Purification

Preparative chromatographic purification was performed using a Gilson PLC 2050 instrument, equipped with a UV detector, and a Waters Delta-Pack 40mm × 100 mm, 100 Å, 15 µm, C18 reversed-phase column using a flow rate of 45 mL/min. Solvents employed were 0.1% aqueous TFA and 0.1% TFA in ACN.

Dynamic Light Scattering and Transmission Electron Microscopy

The size and size distribution of particles were measured using a Nano ZS zetasizer system (Malvern Instruments). The measurement parameters were as follows: a laser wavelength of 633 nm, a scattering angle of 173°, a measurement temperature of 20 °C, a dispersant refractive index of 1.44 and material refractive index of 1.48. Before DLS measurement, the nanoparticles were diluted in DPBS at a concentration of 0.1 mg/mL. The sample was loaded into zetasizer nano series disposable cells and three measurements were performed. The morphologies and dimensions of the samples were revealed with a JEOL 1200 EXII field-emission scanning electron microscope operating at 120kV.

NMR Measurement

Liquid-phase NMR spectra were recorded at room temperature on a BRUKER AVANCE 400MHz (^{19}F) NMR spectrometer in 1.5 M NaOD/D_2O at 25 °C, equipped with a BBFO probe and controlled by TOPSPIN. TFA was chosen as an external standard as it offered the desired signal resolution and adequate solubility properties. Elemental analyses were performed at the Laboratoire de Mesures Physiques (LMP), University of Montpellier, France.

Fluorescence Measurement

Fluorescence measurements were realized on a CLARIOstar from BMG LABTECH (Champigny-sur-Marne, France) with a microplate BMG LABTECH 96. Samples were diluted at 1 mg/mL. The excitation wavelength was 642 nm and the emission wavelength ranged from 670 nm to 710 nm.

1. Synthesis of Hybrid Triethoxysilyl Sulfo Cyanine5.5 for Type D

First, 3.9 milligrams (0.0035 mmol, 1 eq.) of sulfo cyanine5.5 NHS ester was stirred with 115 µL dimethyl sulfoxide-d_6 (DMSO-d_6) and 55 µL (0.23 mmol, 66.5 eq.) 3-aminopropyltriethoxysilane (APTES) for 2 h at room temperature in the dark to obtain hybrid Cyanine 5.5, hereafter called compound **1**, and residual APTES. This solution was used in the next step without further purification

2. Synthesis of Type A, C and D SiNPs

2.1. Type A SiNPs Synthesis

Blank silica nanoparticles were prepared by mixing 1.42 mL (10.2 mmol, 2.25 eq.) ammonia solution at 28% with 25 mL of absolute EtOH. Then, 300 µL DMSO was added, followed by 1 mL TEOS (4.53 mmol, 1 eq.). The reaction mixture was stirred at room temperature for 24 h. Blank SiNPs were precipitated by adding 10 mL absolute EtOH. Blank SiNPs were recovered by centrifugation, washed three times with EtOH (10 mL) and once with DPBS (10 mL), and then conserved in DPBS. Next, 1 mL of the solution was centrifuged, washed twice with water (1 mL) and freeze-dried with 1 mL of water to give a concentration of 9.9 mg/mL.

2.2. Type C SiNPs synthesis

Type C silica nanoparticles were prepared by mixing 465 µL (3.3 mmol, 2.25 eq.) ammonia solution at 28% with 8.2 mL absolute EtOH. The sulfo cyanine5.5 precursor (300 µL, 3 µmol, 3.3 mg) was then added, followed by 328 µL of TEOS (1.48 mmol, 1 eq.). The reaction mixture was stirred at room temperature for 24 h in the dark. After 24 h, 20% mass of TEOS (65 µL, 0.3 mmol) was added to form an extra layer of silica and the reaction mixture was stirred for a further 6 h. Type C SiNPs were precipitated by adding 10 mL absolute EtOH. Type C SiNPs were recovered by centrifugation, washed three times with 10 mL EtOH and three times with 10 mL DPBS, and then conserved in DPBS. Finally, 1 mL of the solution was centrifuged, washed twice with water (1 mL) and freeze-dried with 1 mL of water to give a concentration of 4.8 mg/mL

2.3. Type D SiNPs Synthesis

Type D silica nanoparticles were prepared by mixing 550 µL (3.95 mmol, 2.25 eq.) ammonia solution at 28% with 9.675 mL absolute EtOH. The sulfo cyanine5.5 precursor (300 µL, 3.5 µmol, 3.9 mg) was then added, followed by 387 µL TEOS (1.75 mmol, 1 eq.). The reaction mixture was stirred at room temperature for 24 h in the dark. Type D SiNPs were precipitated by adding absolute 10 mL EtOH. Type D SiNPs were recovered by centrifugation, washed three times with 10 mL EtOH and three times with DPBS (10 mL), and then conserved in DPBS. Finally, 1 mL of the solution was centrifuged, washed twice with water (1 mL) and freeze-dried with 1 mL of water to give a concentration of 7.4 mg/mL.

3. Synthesis of Different Hybrids PEGs

3.1. Synthesis of Fluorinated PEG

Boc-NH-PEG_{2000}-COOH (450 mg, 0.22 mmol, 1eq) was placed in a 100 mL round bottom flask with 20 mL DMF. To this solution were successively added DIEA (0.153 mL, 0.88 mmol, 4eq), 2-fluoroethylamine hydrochloride (29 mg, 0.44 mmol, 2eq) and HATU (166 mg, 0.44 mmol, 2eq). After 1 h of stirring at room temperature, the yellow solution was concentrated under high-vacuum, precipitated by adding cold Et_2O, centrifuged and washed three times with Et_2O. The crude product was dried under vacuum to give 675 mg of a crude compound.

Crude Boc-NH-PEG_{2000}-CH_2CH_2F was purified by preparative HPLC using the following conditions. Nonlinear gradient was run from [100:0, (0.1% aqueous TFA: 0.1% TFA in ACN)] to 100:0 in 5 min, then 100:0 to 75:25 in 3 min, and 75:25 to 55:45 in 20 min with

UV detection at 214 nm. After lyophilization, 0.13 mmol of purified compound was obtained (61% yield).

Boc-NH-PEG_{2000}-CH_2CH_2F was placed in a 100 mL round bottom flask with 20 mL of TFA/DCM (50/50; v/v) solution. After 1 h of stirring at room temperature, the solution was concentrated under high-vacuum, precipitated by adding cold Et_2O, centrifuged and washed three times with Et_2O. The crude compound was then dissolved in water/acetonitrile solution 1/1 v/v and freeze-dried to yield 255.4 mg of fluorinated PEG, which was used without further purification.

3.2. Hyrbid PEGs

To a solution of fluorinated PEG (0.056 mmol, 1 eq.) in 3 mL DMF was added DIEA (0.23 mmol, 4.1 eq.) and (3-isocyanatopropyl)triethoxysilane (0.11 mmol, 2 eq). The reaction mixture was stirred for 2 h at room temperature. After 120 min, DMF was concentrated under high-vacuum and cold 30 mL ether was poured into the reaction mixture to cause precipitation. The precipitate was suspended in ether again and recollected by centrifugation. This procedure was repeated three times to remove ICPTES and DIEA. The crude compound was dried under vacuum and used without further purification to afford compound **2** (0.057mmol, 101% yield).

Compounds **2′**, **3** and **4** were obtained in the same way, using, respectively, trimethylsilylpropyl isocyanate, methyl(diethoxysilyl)propyl isocyanate and dimethyl(chlorosilyl)propryl isocyanate.

4. Synthesis of Hybrid Peptides and Experimental Protocols

4.1. Protocol 1

(i) Fmoc-Gly-OH (5 eq), DIEA (10 eq), DCM, 6 h, rt, SPPS; (ii): deprotection: Pip/DMF (20/80); (iii) coupling: Fmoc-AA-OH (5 eq), HATU (5 eq), DIEA (10 eq), DMF, rt; (iv) TFE:AcOH:DCM (20:10:70, v/v/v), 1h, rt; (v) DIEA (3 eq), HATU (1.1 eq), 4h, rt; (vi) H_2, 10% Pd/C, EtOH, overnight; (vii) Linker (1eq), DIEA (3 eq), HATU (1.1 eq), DMF, 2h, rt; (viii) TFA/TIS/H_2O (95/2.5/2.5), 1 h, rt; (ix) Boc-NH-PEG_{2000}-COOH (1 eq), Cyclic peptide **5** (1 eq), HATU (1.1 eq), DIEA (4.1 eq), 2 h, rt; (x) TFA/DCM (50/50, v/v), 1 h, rt; (xi) 3-isocyanatopropyl triethoxysilane (ICPTES, 2 eq), DIEA (4.1 eq), DMF, 2 h, rt.

(i) Fmoc-AA-OH Anchoring on 2 Chloro Trityl Resin

First, 2-chloro chloro trityl resin (1.6 mmol/g, 1eq) was swelled for 10 minutes in DCM. After DCM removal, the resin was placed in a syringe. Fmoc-AA-OH (5 eq) was anchored to the resin in the presence of DIEA (10 eq) in DCM and stirred for 6 h. After standard washing steps using the following solvents (3 × DCM, 3 × MeOH), capping was performed. The resin was swelled in MeOH with few drops of DIEA and stirred for 30 min. After following step (3 x MeOH, 3 x DCM), Fmoc-AA-Cltrityl-resin was dried under vacuum for 12 h. Fmoc titration was performed.

For *Fmoc-Gly-Cltrityl-resin*, a loading of 0.80 mmol/g was obtained.

For *Fmoc-βAla-Cltrityl-resin*, a loading of 1.07 mmol/g was obtained.

(ii) Standard Fmoc Deprotection

Fmoc-peptidyl-resin was placed in a syringe. The Fmoc group was removed by two successive DMF-piperidine (80:20; v/v) treatments (2 × 2 min). Between each treatment, the solution was filtered off and replaced with a new one.

(iii) Standard Protected Aminoacid Coupling Step

The appropriate volume of the Fmoc-protected amino acid solution (0.5 M) was added to the free N-terminus peptide resin, followed by DIEA (10 eq) and an appropriate volume of HATU solution (0.5 M). The resin was stirred twice for 5 min at rt; after the first

stirring, the solution was filtered off and replaced by a new one. Washing was performed using the following solvents (3× DMF, 3× DCM).

The peptidyl resin Fmoc-Asp(OtBu)-DPhe-Lys(Z)-Arg(Pbf)-Gly-resin was synthesized using standard deprotection and coupling cycles (ii) and (iii). The amino acids used were, successively, Fmoc-Arg(Pbf)-OH, Fmoc-Lys(Z)-OH, Fmoc-(D)Phe-OH, Fmoc-Asp(OtBu)-OH.

The peptidyl resin Boc-βAla-Ala(CF_3)-(βAla)$_2$-resin were synthesized using standard deprotection and coupling cycles (ii) and (iii). The amino acids used were Fmoc-βAla-OH, Boc-βAla-OH and (S)-Fmoc-2-amino-4,4,4-trifluoro-butyric acid.

(iv) Selective Mild Acidic of Peptidyl Resin

Resin was treated with TFE/AcOH/DCM (20/10/70, v/v/v) solution at room temperature for 1 h, filtered and washed twice with cleavage solution. The filtrate was evaporated to dryness under vacuum, and crude peptide was precipitated by addition of cold diethylether. The protected peptide was thus obtained by centrifugation.

After solubilization of crude peptide in a water/acetonitrile 1/1 v/v solution and freeze drying, the partially deprotected peptide was analyzed by LC/MS.

Protected peptide H-Asp(OtBu)-DPhe-Lys(Z)-Arg(Pbf)-Gly-OH was obtained with 67% yield and used without further purification.

Protected peptide Boc-β-Ala-Ala(CF_3)- βAla$_2$-OH was obtained with 49% yield after purification.

(v) Head-to-Tail Cyclization

Protected peptide H-Asp(OtBu)-DPhe-Lys(Z)-Arg(Pbf)-Gly-OH (574.2 mg, 0.54 mmol, 1eq), was placed in a 100 mL round-bottom flask with 15 mL DMF. To this solution were successively added DIEA (0.281 mL, 1.61 mmol, 3eq) and HATU (205 mg, 0.54 mmol, 1 eq). After 4 h stirring at room temperature, the yellow solution was concentrated under high-vacuum, precipitated by adding cold Et_2O, centrifuged and washed three times with Et_2O. The crude compound was then dissolved in a water/acetonitrile solution 1/1 v/v and freeze-dried to yield 761.8 mg of protected cyclic peptide used without purification.

(vi) Benzyloxycarbonyl (Z) Removal in Solution

First, 761.8 mg (0.73 mmol) of cyclic peptide were poured into a 500 mL round-bottom flask fitted with a hole-stopper suitable for bubbling. After solubilization in 150 mL EtOH, a palladium on carbon (10% wt, 77 mg, 0.1 eq) catalyst was added to the solution. The heterogeneous mixture was submitted to hydrogen bubbling under strong magnetic stirring for 24 h at room temperature. The catalyst was filtered out on celite and the filtrate was evaporated under vacuum, affording cyclic peptide (c[Asp(OtBu)-DPhe-Lys-Arg(Pbf)-Gly]) as a white powder which was purified by preparative HPLC using the following conditions. Nonlinear gradient was run from [100:0, (0.1% aqueous TFA: 0,1% TFA in ACN)] to 65:35 in 5 min, and then 65:35 to 45:55 in 20 min with UV detection at 214 nm. After lyophilization, the cyclized compound was obtained with 45% yield.

(vii) Standard Protected Aminoacid Coupling Step in Solution

Cyclic peptide (0.12 mmol, 1eq) was placed in a 50 mL round-bottom flask with 10 mL of DMF. To this solution were successively added DIEA (0.44 mmol, 4eq) and a solution of linker (0.12 mmol, 1eq) mixed with DIEA (0.44 mmol, 4eq.) and HATU (0.12 mmol, 1.1eq.), added drop by drop. After 1 h stirring at room temperature, the solution was concentrated under high-vacuum, precipitated by added cold Et_2O, centrifuged and washed three times with Et_2O. The crude compound was then dissolved in a water/acetonitrile solution 1/1 v/v and freeze-dried.

Crude protected-cyclopeptide **5** c[Asp(OtBu)-DPhe-Lys(Boc-βAla-Ala(CF_3)-βAla$_2$)-Arg(Pbf)- Gly] was obtained (0.11 mmol) and used without further purification (yield 96%).

(viii) Acido Labile Protecting Group Removal

Protected cyclic peptide was poured into a 50 mL round-bottom flask and then treated with a TFA/TIS/H_2O (95/2.5/2.5, v/v/v, 20 mL) solution at room temperature for 1 h. The mixture was evaporated to dryness under vacuum, and crude peptide was precipitated by addition of 30 mL cold diethylether and purified by preparative HPLC.

Cyclopeptide c[Asp-DPhe-Lys(H-βAla-Ala(CF_3)-βAla$_2$)-Arg-Gly] was purified by preparative HPLC using the following conditions. Nonlinear gradient was run from [100:0, (0.1% aqueous TFA: 0.1% TFA in ACN)] to 100:0 in 5 min, then 100:0 to 80:20 in 5 min, and 80:20 to 60:40 in 20 min with UV detection at 214 nm. After lyophilization, 0.068 mmol of deprotected compound **5** was obtained (64% yield).

(ix) Pegylation Step

To a solution of Boc-NH-PEG$_{2000}$-COOH (0.013 mmol, 1 eq.) in 5 mL DMF was added DIEA (4.1 eq.) and HATU (1.1 eq.). The reaction mixture was stirred for 5 minutes to activate the carboxylic acid. Then, the cyclic peptide (0.013 mmol, 1 eq.) was added and the mixture was stirred for 2 h at room temperature. After 2 h, DMF was concentrated under high-vacuum and 30 mL cold ether was poured into the reaction mixture to cause precipitation. The precipitate was suspended in ether again and recollected by centrifugation. This procedure was repeated three times. The crude compound was dried under vacuum, analyzed by analytical LC/MS and used without further purification.

(x) Boc Deprotection

The crude compound was poured into 20 mL of a solution of TFA/DCM (50/50). The mixture was stirred for 1 h at room temperature. The solution was then evaporated under vacuum and 30 mL cold ether was poured into the reaction mixture to cause precipitation. The precipitate was suspended in ether again and recollected by centrifugation. This procedure was repeated three times and the crude product was purified by preparative HPLC.

Cyclopeptide pegylated c[Asp-DPhe-Lys(NH_2-PEG$_{2000}$-βAla-Ala(CF_3)-βAla$_2$)-Arg-Gly] was purified by preparative HPLC using the following conditions. Nonlinear gradient was run from [100:0, (0.1% aqueous TFA: 0.1% TFA in ACN)] to 100:0 in 5 min, then 100:0 to 70:30 in 5 min, and 70:30 to 50:50 in 20 min with UV detection at 214 nm. After lyophilization, 0.086 mmol of deprotected compound was obtained (69% yield).

(xi) Silylation with ICPTES

To the solution of cyclic pegylated peptide (0.019 mmol, 1 eq.) in 3 mL DMF was added DIEA (4.1 eq.) and (3-isocyanatopropyl)triethoxysilane (2 eq). The reaction mixture was allowed to stir for 2 h at room temperature. After 120 min, DMF was concentrated under high-vacuum and 30 mL cold ether was poured into the reaction mixture to cause precipitation. The precipitate was suspended in ether again and recollected by centrifugation. This procedure was repeated three times to remove ICPTES and DIEA. The crude compound was dried under vacuum and used without further purification.

Hybrid PEG-peptide **9** (98% yield)

4.2. Protocol 2

Protocol 2 was the same as Protocol 1 but with the sequence c[Gly-DPhe-Lys[$(EtO)_3Si(CH_2)_3$NHCO-NH-PEG$_{2000}$-βAla-Ala(CF_3)-βAla$_2$)]-Arg-Glu], yielding peptide **8** and the hybrid PEG-peptide **12**.

4.3 Protocol 3

(i) Deprotection: Pip/DMF (20/80); (ii) coupling: Fmoc-AA-OH (5 eq.), HATU (5 eq.), DIEA (10 eq.), DMF, rt; (iii) Ac_2O/DCM (50/50, v/v), rt; (iv) TFA/TIS/H_2O (95/2.5/2.5, v/v/v), 1h, rt; (v) Boc-NH-PEG_{2000}-COOH (1 eq.), Peptide **6** (1 eq.), HATU (1.1 eq.), DIEA (4.1 eq.), 2 h, rt; (vi) TFA/DCM (50/50, v/v), 1 h, rt; (vii) 3-isocyanatopropyl triethoxysilane (ICPTES, 2 eq.), DIEA (4 eq.), DMF, 2h, rt.

(i) Standard Fmoc Deprotection

See Protocol 1 using Fmoc-Arg(Pbf)-Wang resin (100-200 mesh, 0.64mmol/g).

(ii) Standard Protected Aminoacid Coupling Step

See Protocol 1.

(iii) Standard Capping Step

To the resin was added the appropriate volume of capping solution Ac_2O/DCM (50/50, v/v). The resin was stirred twice for 2 min at rt; after the first stirring, the solution was filtered off and replace by a new one. Then, the solution was filtered off. Washing was performed three times using DCM.

The peptidyl resin H-βAla-Phe(4-F)-$βAla_3$-Ala-Thr-Trp-Leu-Pro-Pro-Arg-resin was synthesized using standard deprotection, coupling cycles and capping (i), (ii) and (iii). After the last amino acid, only deprotection step was performed. The amino acids used were, successively, Fmoc-Pro-OH, Fmoc-Leu-OH, Fmoc-Trp(Boc)-OH, Fmoc-Thr(tBu)-OH, Fmoc-Ala-OH, Fmoc-βAla-OH and Fmoc-Phe(4-F)-OH.

(iv) Acido Labile Cleavage and Deprotection Resin Peptidyl

Resin was treated with TFA/TIS/H_2O (95/2.5/2.5, v/v/v) solution at room temperature for 1 h, filtered and washed twice with cleavage solution. The filtrate was evaporated to dryness under vacuum, and crude peptide was precipitated by addition of cold diethylether. The protected peptide was thus obtained by centrifugation.

After solubilization of the crude peptide in a water/acetonitrile 1/1 v/v solution and freeze drying, the deprotected peptide was analyzed by LC/MS and then purified by HPLC preparative.

Crude protected peptide H-βAla-Phe(4-F)-$βAla_3$-Ala-Thr-Trp-Leu-Pro-Pro-Arg-OH was purified by preparative HPLC using the following conditions. Nonlinear gradient was run from [100:0, (0.1% aqueous TFA: 0.1% TFA in ACN)] to 100:0 in 5 min, then 100:0 to 85:15 in 3 min, and 85:15 to 65:35 in 20 min with UV detection at 214 nm. After lyophilization, 0.05 mmol of purified compound **6** was obtained (50% yield).

(v) Pegylation Step

See Protocol 1.

(vi) Boc Deprotection

See Protocol 1.

(vii) Silylation with ICPTES

See Protocol 1.

Hybrid PEG-peptide **10** (99% yield).

4.4. Protocol 4

(i) Deprotection: Pip/DMF (20/80); (ii) coupling: Fmoc-AA-OH (5 eq.), HATU (5 eq.), DIEA (10 eq.), DMF, rt; (iii) Ac_2O/DCM (50/50, v/v), rt; (iv) TFA/TIS/H_2O (95/2.5/2.5, v/v/v), 1h, rt. (v) Boc-NH-PEG_{2000}-COOH (1 eq.), Peptide **7** (1 eq.), HATU (1.1 eq.), DIEA (4.1 eq.), 2 h, rt; (vi) TFA/DCM (50/50, v/v), 1 h, rt; (vii) 3-isocyanatopropyl triethoxysilane (ICPTES, 2 eq.), DIEA (4.1 eq.), DMF anhy, 2h, rt; (viii) hν 356nm, 3h, DMF.

(i) Standard Fmoc Deprotection

See Protocol 1 using AmphiSpheres resin (40 RAM, 0.35 mmol/g).

(ii) Standard Protected Aminoacid Coupling Step

The appropriate volume of the Fmoc-protected amino acid solution (0.5 M) was added on the free N-terminus peptide resin, followed by DIEA (10 eq.) and an appropriate volume of HATU solution (0.5 M). The resin was stirred twice for 5 min at rt, except for Fmoc-Lys(NVOC)-OH (2 eq.) and Fmoc-Phe(4-CF_3)-OH (2 eq.), for which the resin was stirred once for 5 h or overnight. Washing was performed using the following solvents (3× DMF, 3× DCM).

(iii) Standard Capping Step

See Protocol 2.

(iv) Acido Labile Cleavage and Deprotection Resin Peptidyl

See Protocol 2.

Crude protected peptide H-Phe(4-CF_3)-βAla$_2$-Gly-Thr-Pro-Gly-Lys(NVOC)-Hyp-Gly-Pro-Arg-Gly-Gln-Arg-Gly-Pro-Thr-Gly-Pro-Arg-Gly-Glu-Arg-Gly-Pro-NH_2 was purified by preparative HPLC using the following conditions. Nonlinear gradient was run from [100:0, (0.1% aqueous TFA: 0.1% TFA in ACN)] to 100:0 in 5 min, then 100:0 to 85:15 in 5 min, and 85:15 to 65:35 in 20 min with UV detection at 214 nm. After lyophilization, 0.028 mmol of compound **7** was obtained (28% yield).

(v) Pegylation Step

See Protocol 1.

(vi) Boc Deprotection

See Protocol 1.

(vii) Silylation with ICTPES

See Protocol 1.

(viii) NVOC Deprotection

Hybrid peptide was poured into DMF (15 mg/mL) and stirred for 2 h under a UV lamp (UVA 320-395nm). After 2 h, an orange/brown color of the media was obtained as a result of the nitro side product. DMF was evaporated under high-vacuum and 30 mL cold ether was poured into the round bottom flask to cause precipitation. The precipitate was suspended in ether again and recollected by centrifugation. This procedure was repeated twice. The crude compound was dried under vacuum and used without further purification.

Hybrid peptide **11** (99% yield).

5. Grafting of Hybrid PEG

To achieve grafting of hybrid PEG, compound **2** (16 µmol) was solubilized in 700 µL of a solution of anhydrous DMF/AcOH 1% (v/v), then poured on SiNPs previously centrifuged (2.777 mL of SiNPs solution at 10.8 mg/mL to give 30 mg of fluorescent SiNPs), and free of the supernatant. This solution was correctly resuspended and stirred overnight at 65 °C. Then, 10 mL DMF was added, and the solution was centrifuged at 7800 rpm before the filtrate was removed. This washing procedure was repeated twice with 10 mL DMF, and twice with 10 mL EtOH and 10 mL DPBS. Then, the SiNPs were kept in sus-

pension in 5 mL DPBS. For yield determination by ^{19}F NMR, an 800 µL aliquot of the suspension was centrifuged, washed twice with 1 mL of pure water and then poured in 1 mL of pure water before freeze-dried to give 4.4 mg of SiNP powder.

6. Calculations for Number of PEG/nm2

To achieve the number of PEG or ligand per nm square, we used the volume of the SiNPs sphere and the density of silica. With a radius of 55 nm, the volume was 696909 nm^3 for one SiNP. The density of the silica was 2.2 g/cm^3, so the number of NP/g was 1/(ρ/V), so 6.5 × 10^{14}. From the number of µmol obtained per NMR sample, we could determine the number of µmol of PEG per g, and so the number of PEG (N_{PEG}) per g using Avogadro's constant. With the number of NP per g calculated, we could then determine the number of PEG per Np (N_{PEG}/NP), and thus, the number per nm^2 (N_{PEG}/nm^2) with the sphere area.

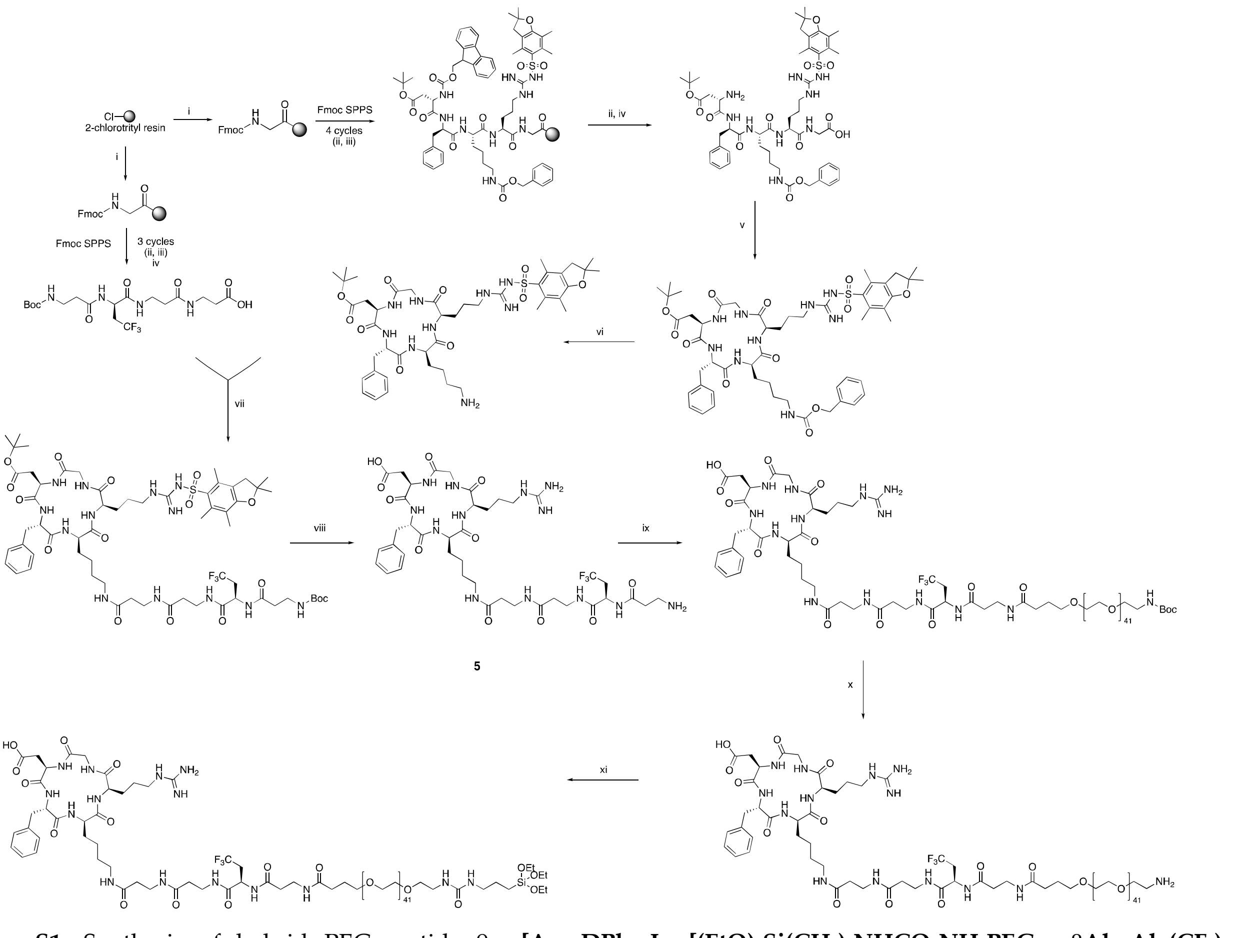

Figure S1. Synthesis of hybrid PEG-peptide 9: **c[Asp-DPhe-Lys[$(EtO)_3Si(CH_2)_3$NHCO-NH-PEG_{2000}-βAla-Ala(CF_3)-$βAla_2$)]-Arg-Gly].**

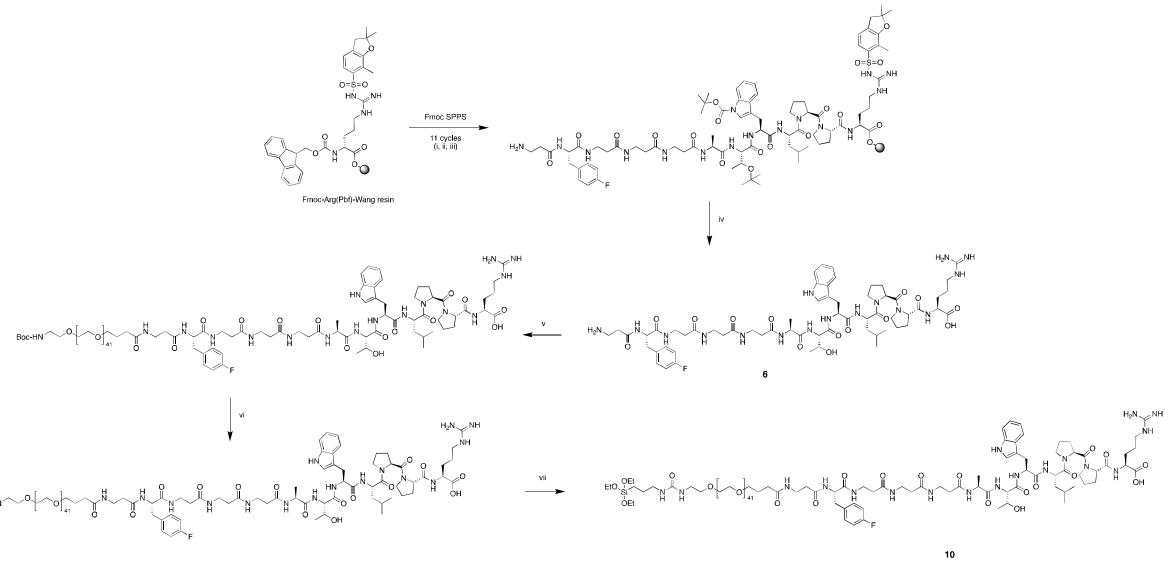

Figure S2. Synthesis of hybrid PEG-peptide **10**: **$(EtO)_3Si(CH_2)_3$NHCO-NH-PEG$_{2000}$-βAla-Phe(4-F)-βAla$_3$-Ala-Thr-Trp-Leu-Pro-Pro-Arg-OH**.

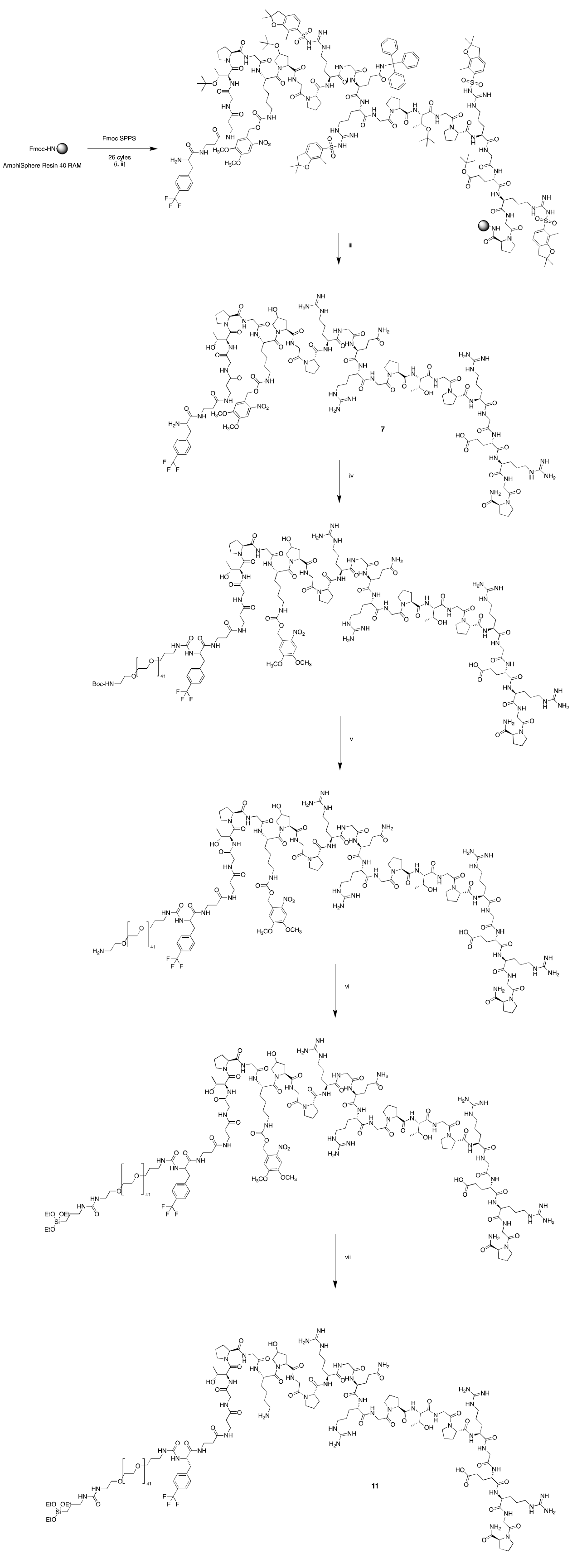

Figure S3. Synthesis of hybrid peptide **11**: **(EtO)$_3$Si(CH$_2$)$_3$NHCOPhe(4-CF$_3$)-βAla$_2$-Gly-Thr-Pro-Gly-Lys-Hyp-Gly-Pro-Arg-Gly-Gln-Arg-Gly-Pro-Thr-Gly-Pro-Arg-Gly-Glu-Arg-Gly-Pro-NH$_2$.**

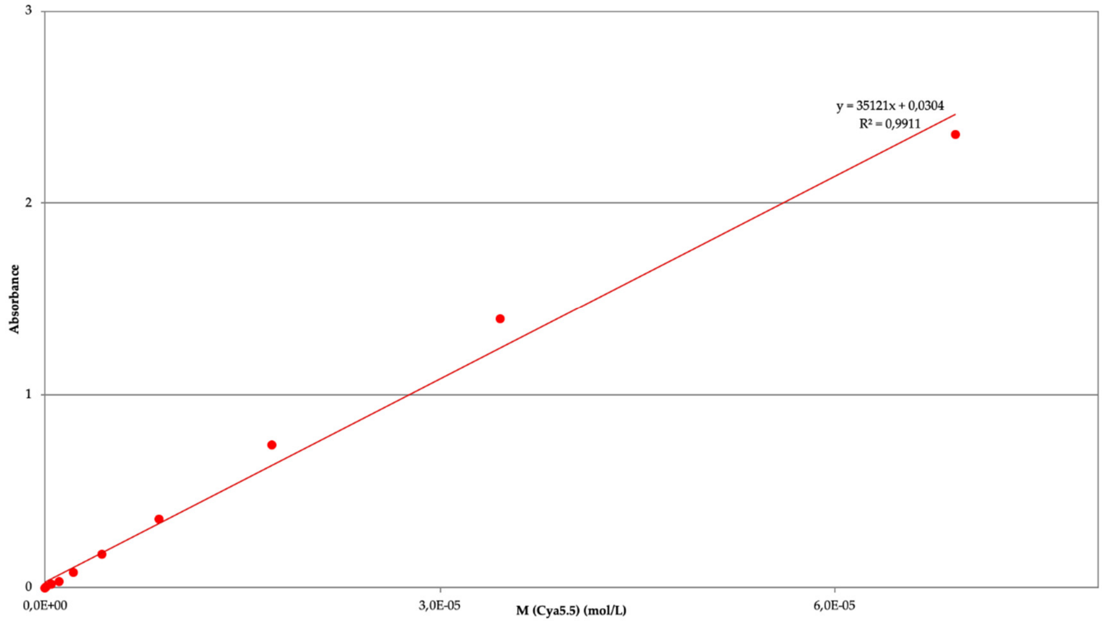

Figure S4. Calibration curve for Cyanine 5.5 encapsulation

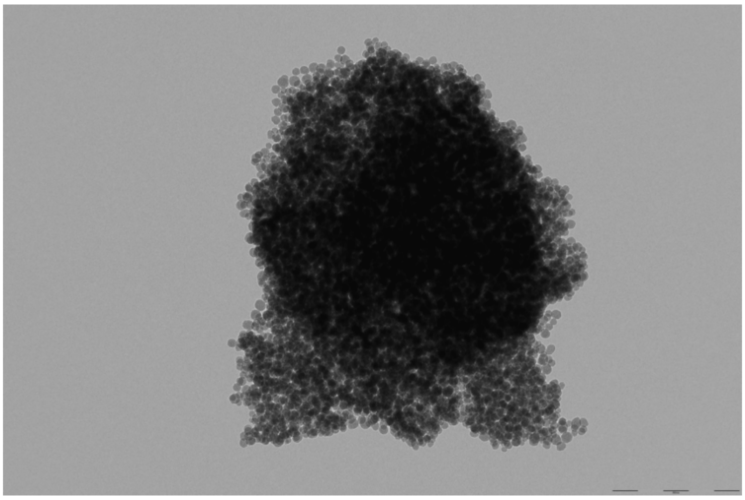

Figure S5. Aggregated dried SiNPs.

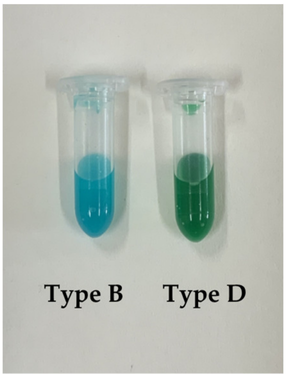

Figure S6. Type **B** vs Type **D** SiNPs.

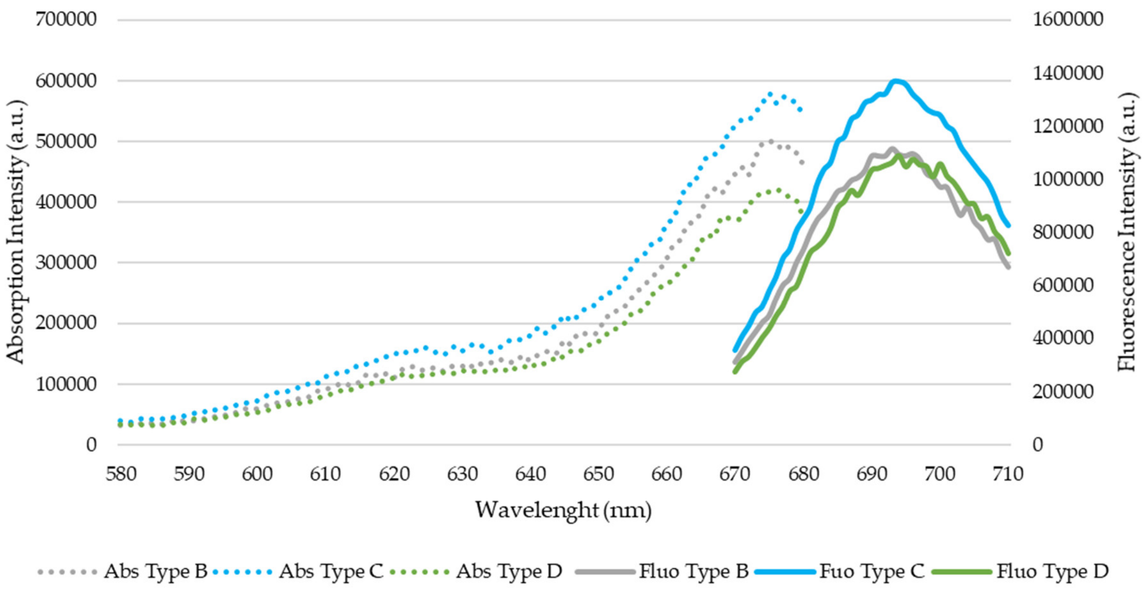

Figure S7. UV/Visible spectra of SiNPs Type B, C and D.

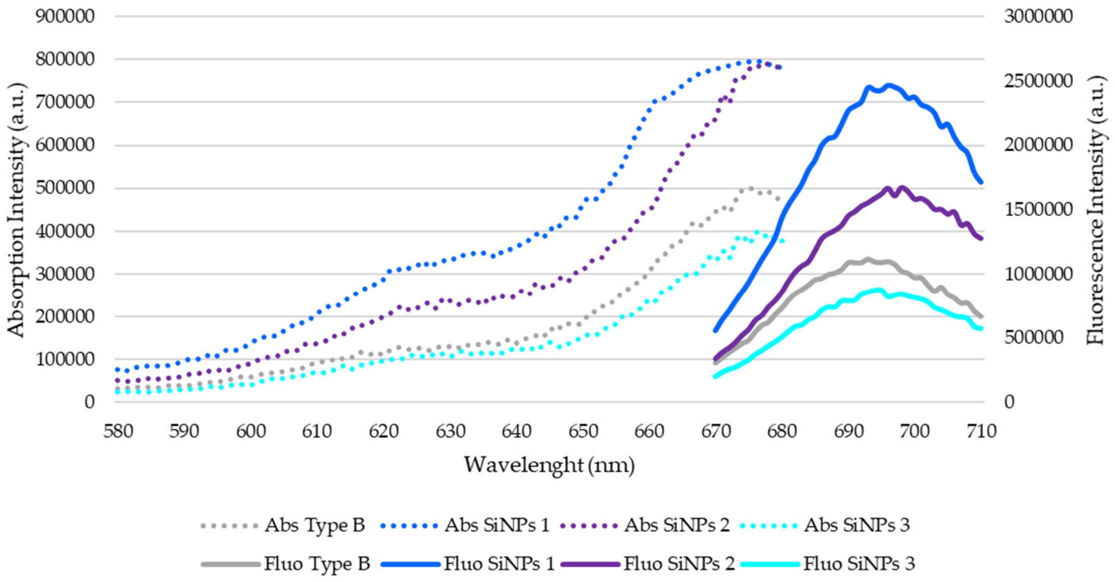

Figure S8. UV/Visible spectra of SiNPs Type B, SiNPs 1, SiNPs 2 and SiNPs 3
